# Supplementary material for: Bayesian traction force estimation using cell boundary-dependent force priors
Source: Biophys J. 2023 Nov 2;122(23):4542–54. doi: 10.1016/j.bpj.2023.10.032 (PMC10719052; doi:10.1016/j.bpj.2023.10.032)
Supplement: Document S1. Supporting methods and Figures S1–S8 [file mmc1.pdf]

Biophysical Journal, Volume *122*

Supplemental information

Bayesian traction force estimation using cell boundary-dependent force priors

Ryosuke Fujikawa, Chika Okimura, Satoshi Kozawa, Kazushi Ikeda, Naoyuki Inagaki, Yoshiaki Iwadate, and Yuichi Sakumura

Supplemental Methods

1. Virtual cell deformation by mean curvature flow

The changes in cell shape using LSF can be described by the Hamilton–Jacobi equation:

$$\frac{\partial \phi(\mathbf{x},t)}{\partial t} = F(\mathbf{x},t;\phi)|\nabla\phi(\mathbf{x},t)|\,, \tag{S1}$$

where $F(\mathbf{x},t;\phi)$ and $|\nabla\phi(\mathbf{x},t)|$ represent the velocity of edge movement and the length of the normal vector at each edge point, respectively. Because edge points are sets that satisfy $\phi(\mathbf{x},t)=0$, increasing $\phi(\mathbf{x},t)$ to $\phi(\mathbf{x},t)>0$ implies cell retraction. Thus, the cell retracts when the velocity $F(\mathbf{x},t;\phi)$ is positive. We defined the velocity $F(\mathbf{x},t;\phi)$ using the mean curvature flow (MCF) algorithm (1). The MCF algorithm diffuses and homogenizes the curvature of a curve or surface. The change of the average curvature at the edge point $\mathbf{x}$ at time t, $\kappa(\mathbf{x},t;\ \phi)$, is defined as

$$\kappa(\mathbf{x},t;\ \phi) = -\frac{1}{2}\nabla\cdot\left(\frac{\nabla\phi(\mathbf{x},t)}{|\nabla\phi(\mathbf{x},t)|}\right), \tag{S2}$$

which is negative at protruding edges and reduces the curvature. We then defined the edge movement velocity as

$$F(\mathbf{x},t;\phi) = -D\kappa(\mathbf{x},t;\ \phi)\,, \tag{S3}$$

where D is the location-independent diffusion coefficient. We substituted Eq. (S3) into Eq. (S1) and moved the edges until time $t=\tau$, when the curvature was sufficiently diffused. In this study, τ was set such that $\tau > 0.1[Cell\ area]\ /\ D$ on the basis of 10% of the cell area. This condition increases τ to diffuse curvature for cells with large areas. We used MATLAB (Mathworks) to simulate cell deformation and Mitchell's program package (2) to calculate the MCF.

2. EM algorithm for estimating forces and hyperparameters

We used the EM algorithm to maximize the log posterior probability distribution $\log P(\{\theta_n,s_n\}_{n=1}^N|\mathbf{u}_{obs})$ of the angle and magnitude $\{\theta_n,s_n\}$ $(n=1,\cdots,N)$ of each force for the bead displacement observation $\mathbf{u}_{obs}$, and simultaneously approximate the posterior probability distribution of traction force $\mathbf{f}$, $P(\mathbf{f}|\mathbf{u}_{obs},\{\theta_n,s_n\}_{n=1}^N)$. Using the likelihood of bead displacement,

$$P(\mathbf{u}_{obs}|\{\theta_n,s_n\}_{n=1}^N) = \frac{P(\mathbf{u}_{obs}|\boldsymbol{f})\times P(\boldsymbol{f}|\{\theta_n,s_n\}_{n=1}^N)}{P(\boldsymbol{f}|\mathbf{u}_{obs},\{\theta_n,s_n\}_{n=1}^N)}\,,$$

where $P(\mathbf{f}|\mathbf{u}_{obs},\{\theta_n,s_n\}_{n=1}^N)$ is $\mathbf{f}$ posterior, we obtained posterior probability distributions for the angle and magnitude of the forces:

$$P(\{\theta_n,s_n\}_{n=1}^N|\mathbf{u}_{obs}) \propto P(\mathbf{u}_{obs}|\{\theta_n,s_n\}_{n=1}^N)\times P(\{\theta_n,s_n\}_{n=1}^N)\,. \tag{S4}$$

Therefore, the log posterior probability distribution of $\{\theta_n,s_n\}$ became

$$\log P(\{\theta_n,s_n\}_{n=1}^N|\mathbf{u}_{obs}) = \log P(\mathbf{u}_{obs}|\{\theta_n,s_n\}_{n=1}^N) + \log P(\{s_n\}_{n=1}^N) + const.\,, \tag{S5}$$

where we included the uninformative direction prior $P(\{\theta_n\})$ in the third term. To estimate the hyperparameters $\{\theta_n,s_n\}$ from Eq. (S5) for the bead displacement observation $\mathbf{u}_{obs}$, we introduced the EM algorithm while obtaining an approximate function to estimate $\mathbf{f}$.

The sum of the first and second terms in Eq. (S5) determines the maximum log-posterior. For the first term, we used a probability density function $q(\mathbf{f})$ that approximates $\mathbf{f}$ posterior $P(\mathbf{f}|\mathbf{u}_{obs},\{\theta_n,s_n\}_{n=1}^N)$ and transformed the expression as follows:

$$
\begin{aligned}
\log P(\mathbf{u}_{obs}|\{\theta_n, s_n\}_{n=1}^N) &= \int_{\mathbb{R}^{2N}} q(\mathbf{f}) \log \frac{P(\mathbf{u}_{obs}|\mathbf{f}) \times P(\mathbf{f}|\{\theta_n, s_n\}_{n=1}^N)}{P(\mathbf{f}|\mathbf{u}_{obs}, \{\theta_n, s_n\}_{n=1}^N)} d\mathbf{f} \\
&= -\int_{\mathbb{R}^{2N}} q(\mathbf{f}) \log \frac{q(\mathbf{f})}{P(\mathbf{u}_{obs}|\mathbf{f}) \times P(\mathbf{f}|\{\theta_n, s_n\}_{n=1}^N)} d\mathbf{f} \\
&\quad + \int_{\mathbb{R}^{2N}} q(\mathbf{f}) \log q(\mathbf{f})\, d\mathbf{f} \\
&\quad - \int_{\mathbb{R}^{2N}} q(\mathbf{f}) \log P(\mathbf{f}|\mathbf{u}_{obs}, \{\theta_n, s_n\}_{n=1}^N)\, d\mathbf{f}\ . \qquad \text{(S6)}
\end{aligned}
$$

The integral in the first term of Eq. (S6) is non-negative and is known as the Kullback–Leibler divergence; it is zero when $q(\mathbf{f}) = P(\mathbf{u}_{obs}|\mathbf{f}) \times P(\mathbf{f}|\{\theta_n, s_n\}_{n=1}^N)$. On the basis of our EM algorithm, we alternately update the **f** posterior approximation $q(\mathbf{f})$ and $\{\theta_n, s_n\}_{n=1}^N$ to maximize Eq. (S5) while satisfying this condition. Let the value of $\{\theta_n, s_n\}$ at the k-th update be written as $\{\theta_n^k, s_n^k\}$. In the expectation step (E-step), $\{\theta_n^k, s_n^k\}$ is fixed and $q(\mathbf{f})$ is approximated by a Gaussian distribution:

$$
q\left(\mathbf{f}\middle|\boldsymbol{\mu}_f^k, \Sigma_f\right) \propto \exp\left\{-\frac{1}{2}\left(\mathbf{f} - \boldsymbol{\mu}_f^k\right)^T \Sigma_f^{-1}\left(\mathbf{f} - \boldsymbol{\mu}_f^k\right)\right\}. \qquad \text{(S7)}
$$

The mean $\boldsymbol{\mu}_f$ and covariance Σ_f of the parameters are the values for which the first term in Eq. (S6) is zero; we therefore obtained

$$
\begin{aligned}
\boldsymbol{\mu}_f^k &= \Sigma_f\left(\alpha G^T \mathbf{u}_{obs} + \beta \mathbf{f}_{\mu}\{\theta_n^k, s_n^k\}\right) \\
\Sigma_f &= (\alpha G^T G + \beta I_{2N})^{-1},
\end{aligned}
$$

where the parameters and the variables are explained in the text. In the maximization step (M-step), when the approximated distribution $q\left(\mathbf{f}\middle|\boldsymbol{\mu}_f^k, \Sigma_f\right)$ is substituted into the log-likelihood (Eq. (S6)), the k-th log-posterior distribution (Eq. (S5)) becomes

$$
Q^k(\{\theta_n, s_n\}_{n=1}^N) = -\int_{\mathbb{R}^{2N}} q\left(\mathbf{f}\middle|\boldsymbol{\mu}_f^k, \Sigma_f\right) \log P(\mathbf{f}|\mathbf{u}_{obs}, \{\theta_n, s_n\}_{n=1}^N)\, d\mathbf{f} - \gamma \sum_{n=1}^{N} |s_n|\ . \qquad \text{(S8)}
$$

We found $\{\theta_n^k, s_n^k\}$ that maximizes this distribution. By repeating the E- and M-steps, we incrementally computed

$$
\begin{aligned}
\mathbf{f}_{est} &= \arg\max_{\mathbf{f}} q\left(\mathbf{f}\middle|\boldsymbol{\mu}_f^k, \Sigma_f\right) \\
\{\theta_n^{k+1}, s_n^{k+1}\} &= \arg\max_{\{\theta_n, s_n\}} Q^k(\{\theta_n, s_n\}_{n=1}^N)\,. \qquad \text{(S9)}
\end{aligned}
$$

We then adopted $\{\theta_n, s_n\}_{n=1}^N$ when $\mathbf{f}_{est}$ was sufficiently convergent. The initial conditions were $\theta_n^0 = 0$, $s_n^0 = 1$, $(n = 1, \dots, N)$. A conceptual diagram illustrating the prior design characterized by hyperparameters and the EM algorithm is provided in **Fig. S1***B*.

3. Software for quantifying cells and estimating forces

We developed MATLAB software for force estimation using Bayesian traction force estimation (BTFE), ridge, and lasso regressions. The software, available at https://github.com/sakulab-software/Bayesian_Force_Estimation, provides a graphical interface for viewing and manipulating microscopic images. Users can easily perform image preprocessing and extract cellular regions using intuitive tools. The software requires the MATLAB main unit and Image Processing Toolbox (version R2021a or later) and supports computational efficiency through the Parallel Computing Toolbox.

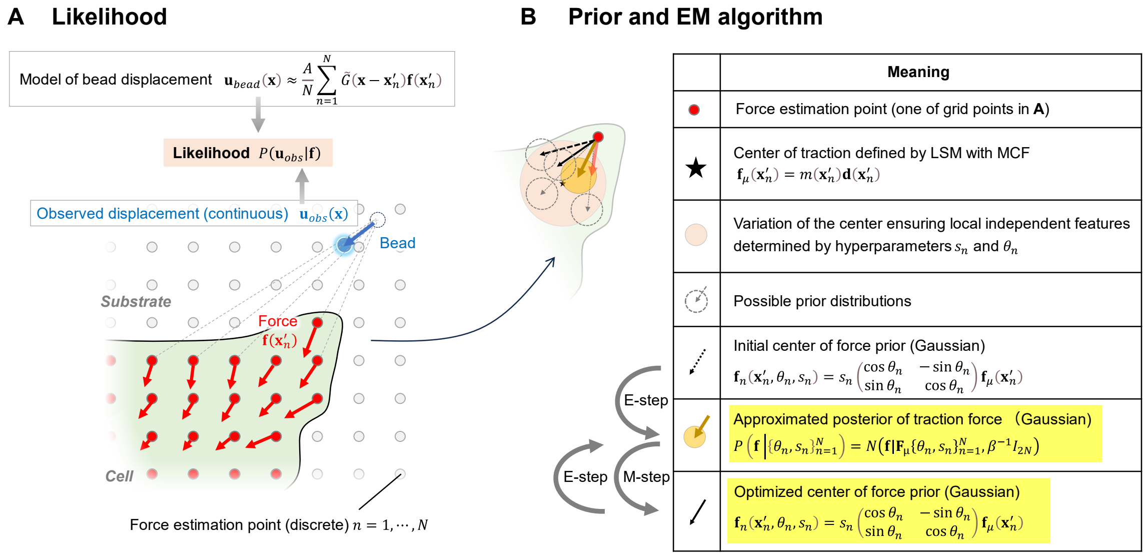

Figure S1. Conceptual diagram of likelihood, prior, and approximation algorithm

(A) The likelihood is calculated on the basis of the difference between the observed continuous bead displacement $\mathbf{u}_{obs}$ (blue vector) and the model displacement $\mathbf{u}_{bead}$ resulting from the force $\mathbf{f}$ arranged on a grid. The force $\mathbf{f}$ at each grid point (red arrow) is the estimation target.

(B) The relationship between the design of the prior and the EM algorithm used for approximating the force $\mathbf{f}$. Initially, the center of the force prior ($\mathbf{f}_{\mu}$) at each grid point (red point) is roughly determined from the cell boundary in terms of magnitude (m) and direction ($\mathbf{d}$) (black star; refer to the Methods section in the main text); $\mathbf{f}_{\mu}$ is locally scaled (s) and rotated (θ) at each grid point to create the actual center of the prior ($\mathbf{f}_n$). Candidates for $\mathbf{f}_n$ exist within the pink filled circle, and their surrounding area (gray dashed edge circle) represents the distribution of the force prior. Parameters s and θ are iteratively adjusted to maximize the peak of the posterior distribution of $\mathbf{f}$ (maximum a posteriori; MAP, gold arrow). Initially, an initial value (dotted arrow) is set, and the peak $\mathbf{f}$ of $\mathbf{f}$'s posterior distribution is calculated (E-step, Eq. (S7)). With the force fixed at that peak, s and θ are then optimized using MAP estimation (solid black arrow; M-step, Eq. (S8)). This process is repeated for each grid point until convergence.

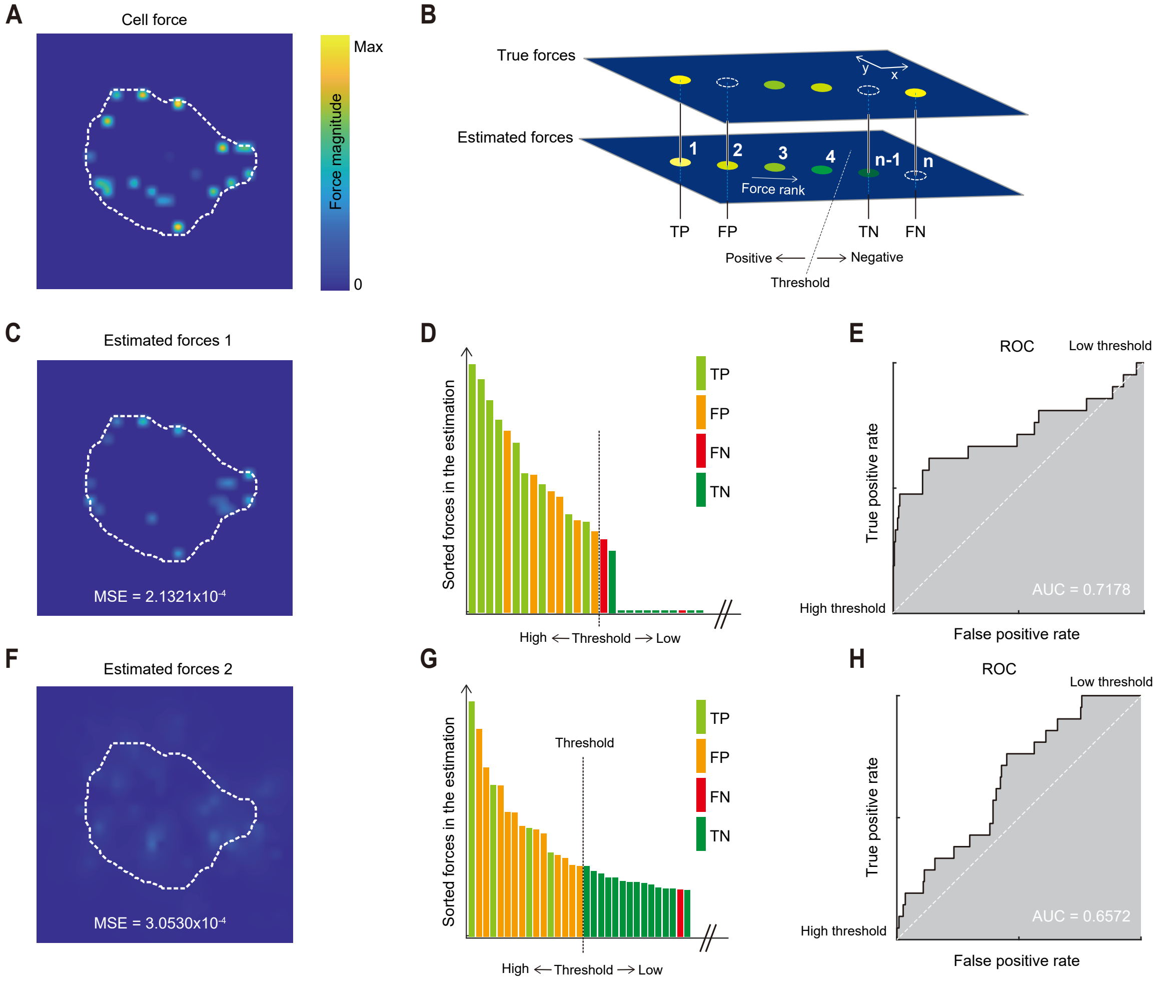

Figure S2. Derivation of ROC curves for evaluating force estimation accuracy.
(A) Force distribution generated by the model cell, with the force magnitude represented by a color bar.
(B) Threshold value used to determine positive and negative force detection. A force greater than the threshold was considered positive, whereas one less than the threshold was deemed negative. Determination of true or false was based on the presence or absence, respectively, of model cell forces. We calculated the rates of true positives and false positives for each threshold and drew ROC curves.
(C) Sample image of the forces estimated by the Bayes approach.
(D) Bar graph of the forces in (C), sorted by magnitude, with the four labels displayed in different colors.
(E) ROC curve derived from (D). The true positive rate is calculated as TP/(TP + FN), and the false positive rate is determined as FP/(FP + TN). Both the true positive rate and the false positive rate are low when the thresholds are large because only large estimated forces are positive. Decreasing the threshold raises the false positive force due to noise while capturing more true positive force.
(F–H) Same as (C–E) but with the forces estimated by ridge regression.

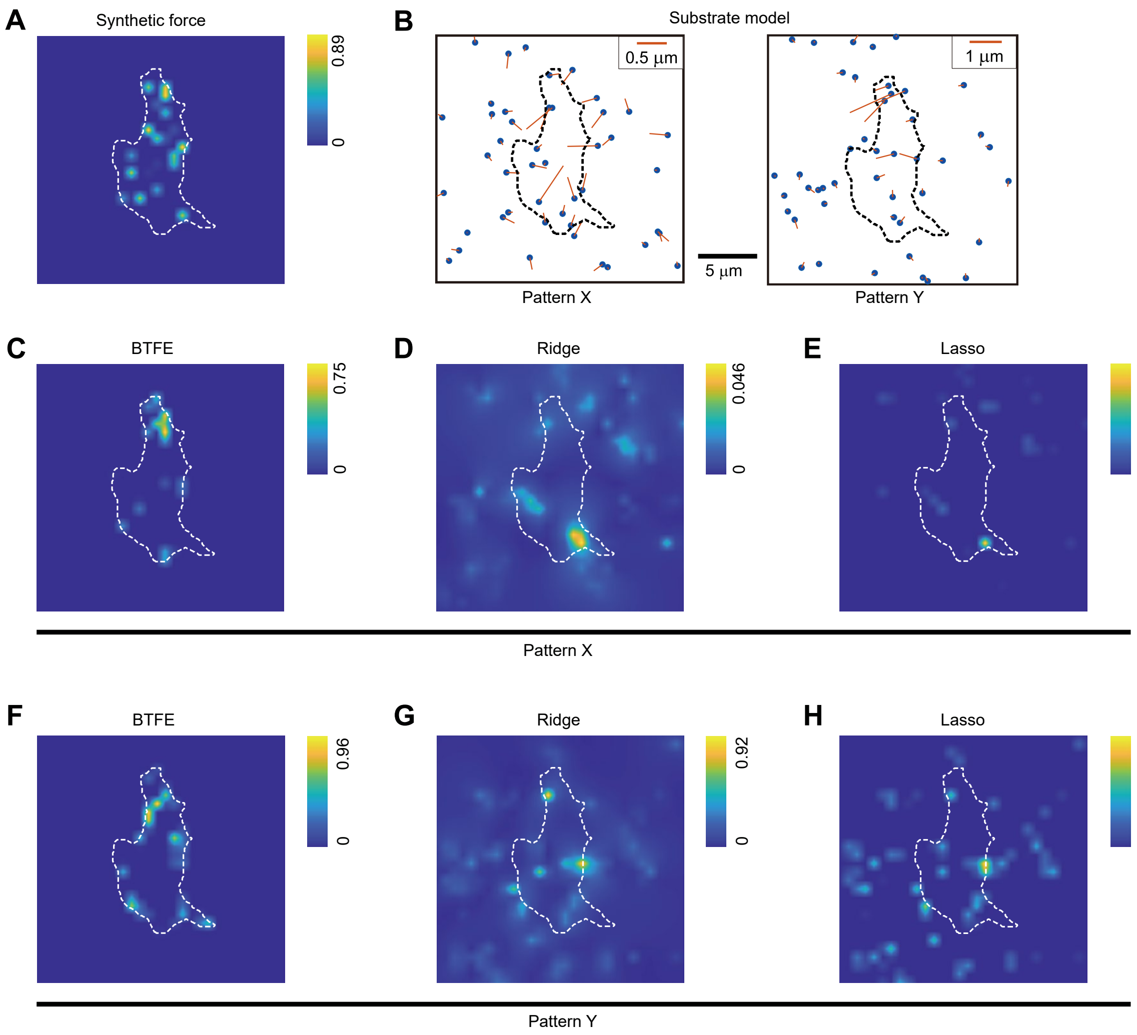

Figure S3. Dependence of force estimation on bead location for bead density of 0.4 beads/µm^2.

(A) The synthetic force in Fig. 4*A*, redisplayed for visual comparison with estimated forces.

(B) Two patterns of bead locations randomly spread at 0.4 bead/µm^2 on the substrate, different from Fig. 4*B*.

(C–E) Comparison of estimation results from the three different algorithms (C, Bayes; D, ridge; E, lasso) for the case of pattern X bead allocation.

(F–H) Estimation results for the case of pattern Y bead allocation.

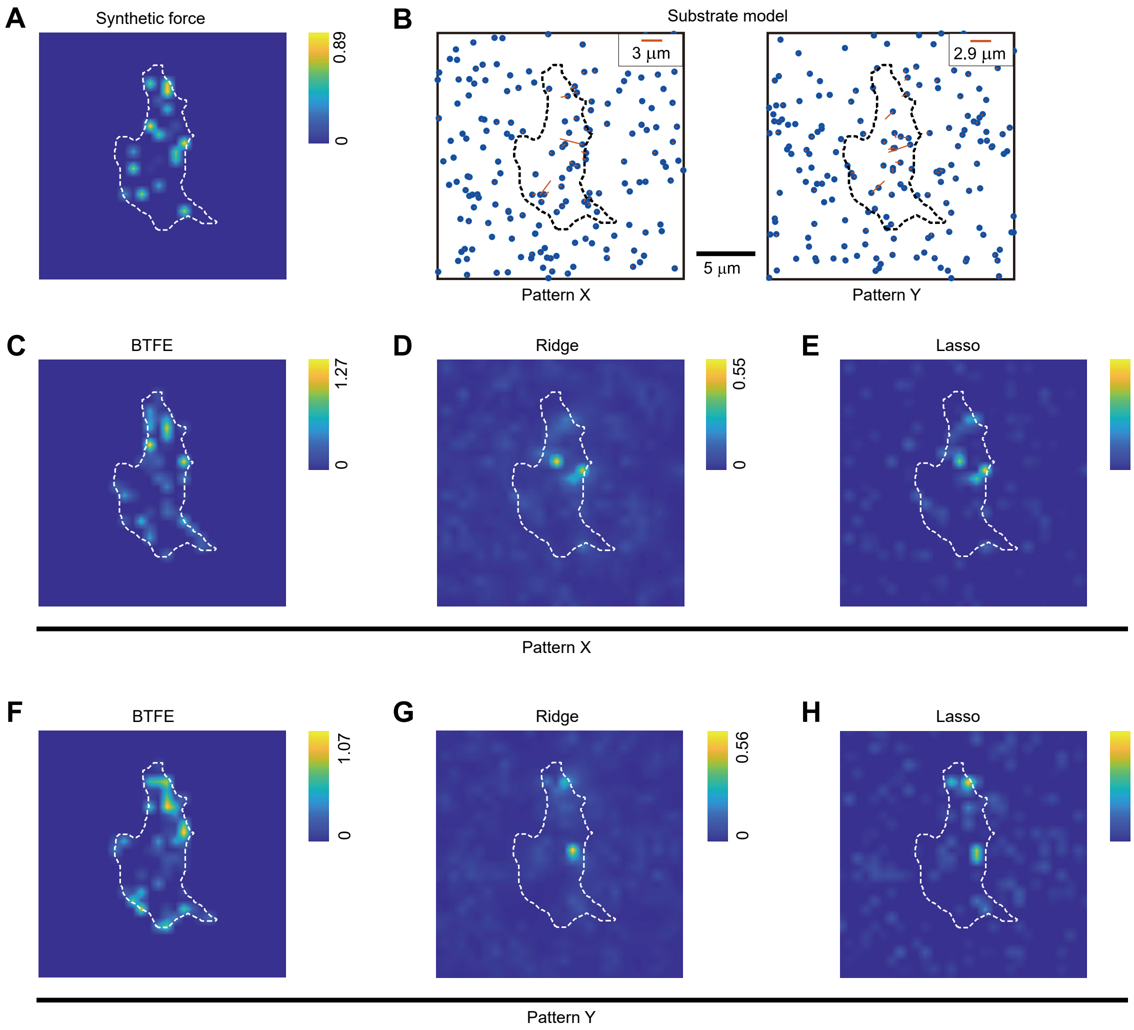

Fig. S4. Dependence of force estimation on bead location for bead density of 1.6 beads/µm^2.

(A) The synthetic force in **Fig. 4***A*, redisplayed for visual comparison with estimated forces. (B) Two patterns of bead positions randomly spread at 1.6 bead/µm^2 on the substrate, different from **Fig. 4***B*. (C–E) Comparison of estimation results from the three different algorithms (C, Bayes; D, ridge; E, lasso) for the case of pattern X bead allocation.

(F–H) Estimation results for the case of pattern Y bead allocation.

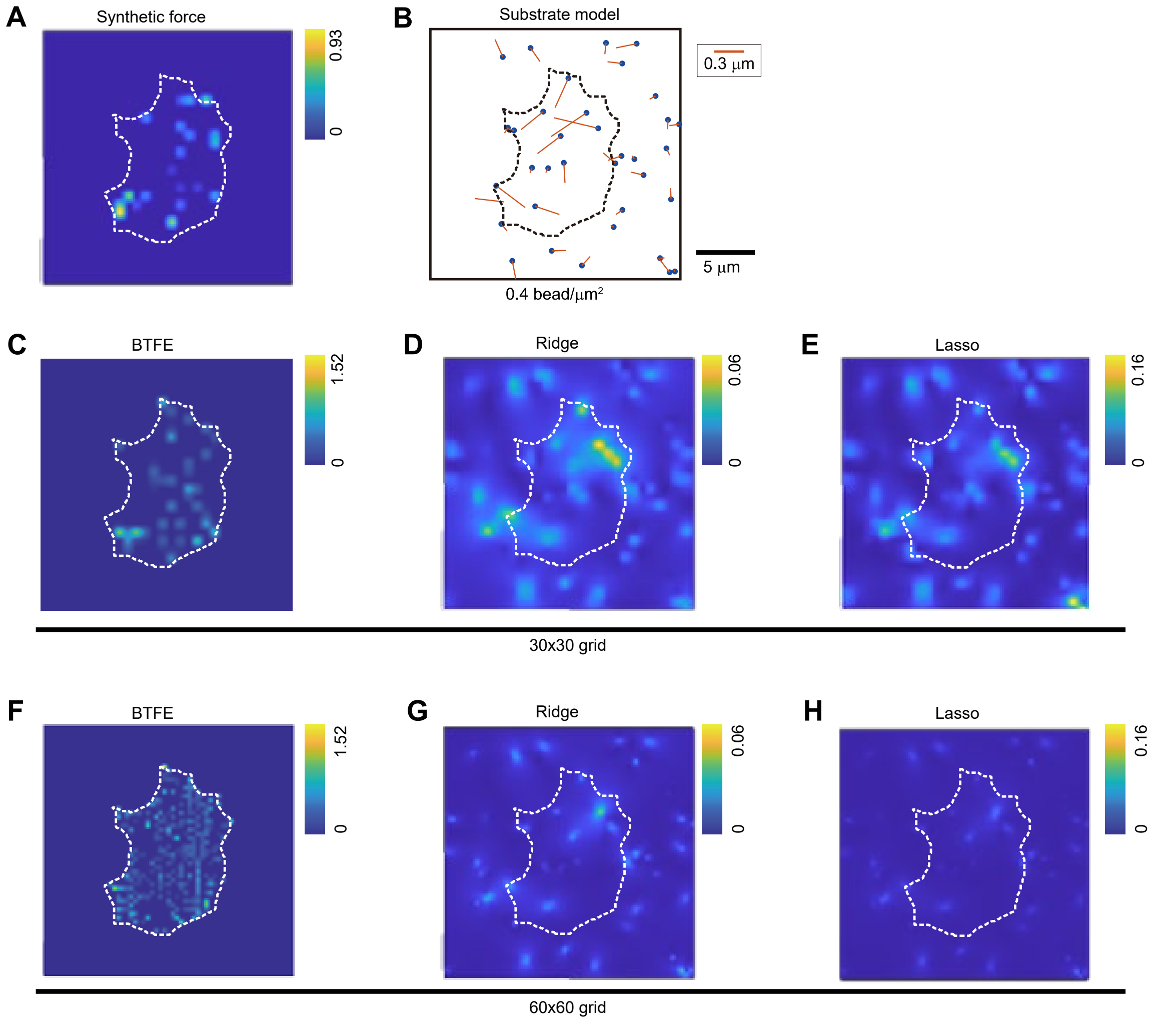

Figure S5. Dependency of force estimation on grid density.
(A) The synthetic force.
(B) Bead positions randomly distributed at 0.4 bead/μm^2 on the substrate.
(C)–(E) Estimation results calculated by BTFE, ridge, and lasso, respectively, using the default grid density (30×30).
(F)–(H) Corresponding results using a high grid density (60×60).

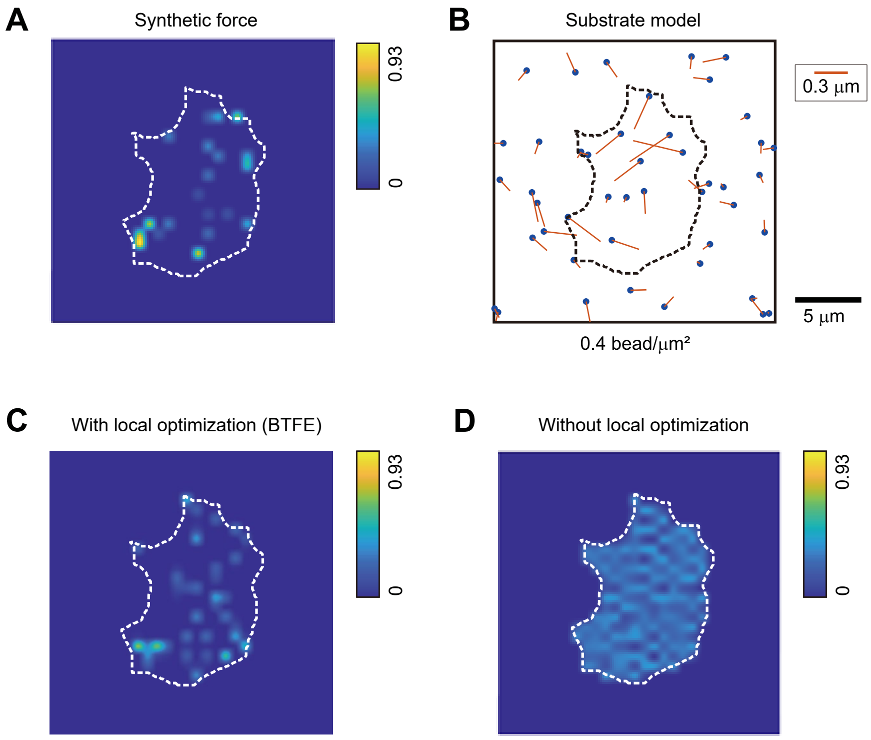

Figure S6. Effect of local optimizations to force prior on force estimation accuracy.
(A) Assumed artificial force generation based on the boundary of the HT1080 cell.
(B) Bead displacement resulting from the force in (A).
(C) Estimation results using locally optimized force priors in BTFE.
(D) Estimation results using force priors without local optimization.

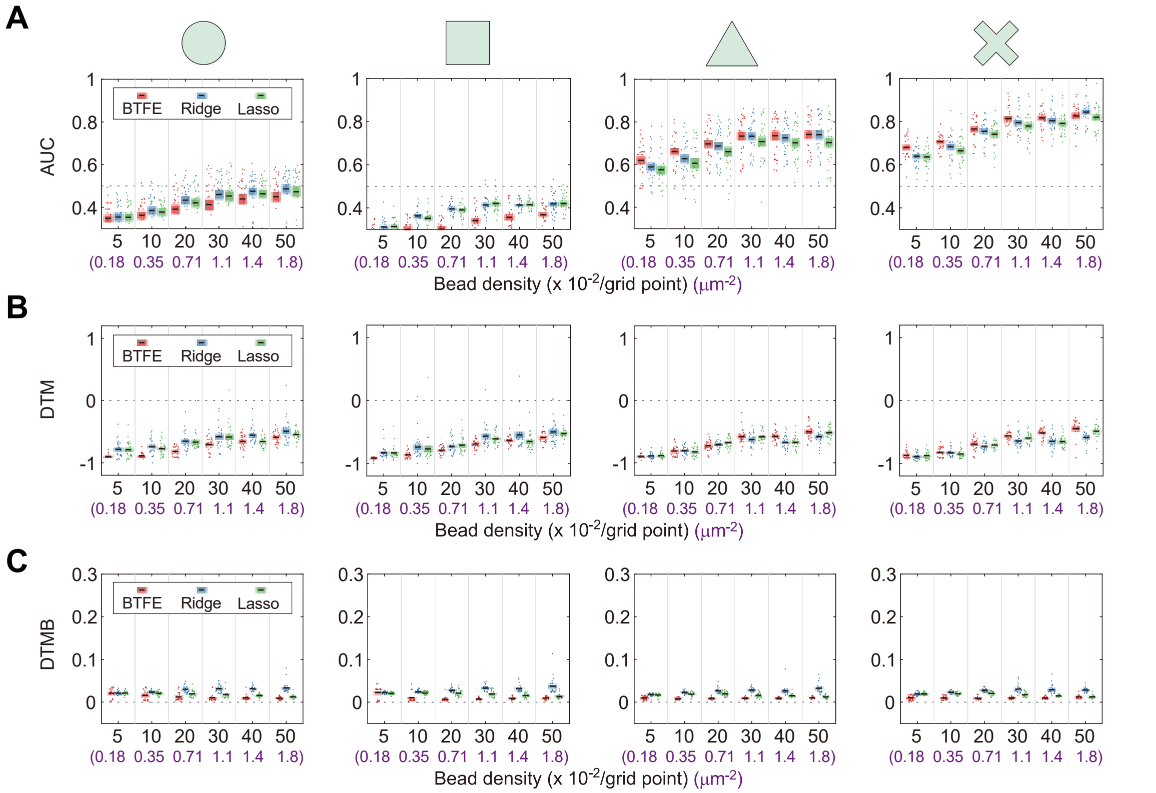

Figure S7. Relationship between geometric boundaries and force estimation accuracy. From left to right, we evaluated force estimation for cells assumed to have the boundaries of a perfect circle, square, equilateral triangle, and X-shape. Metrics used for evaluation include AUC (A), DTM (B), and DTMB (C), following the format outlined in **Figs. 5***D*–*F*.

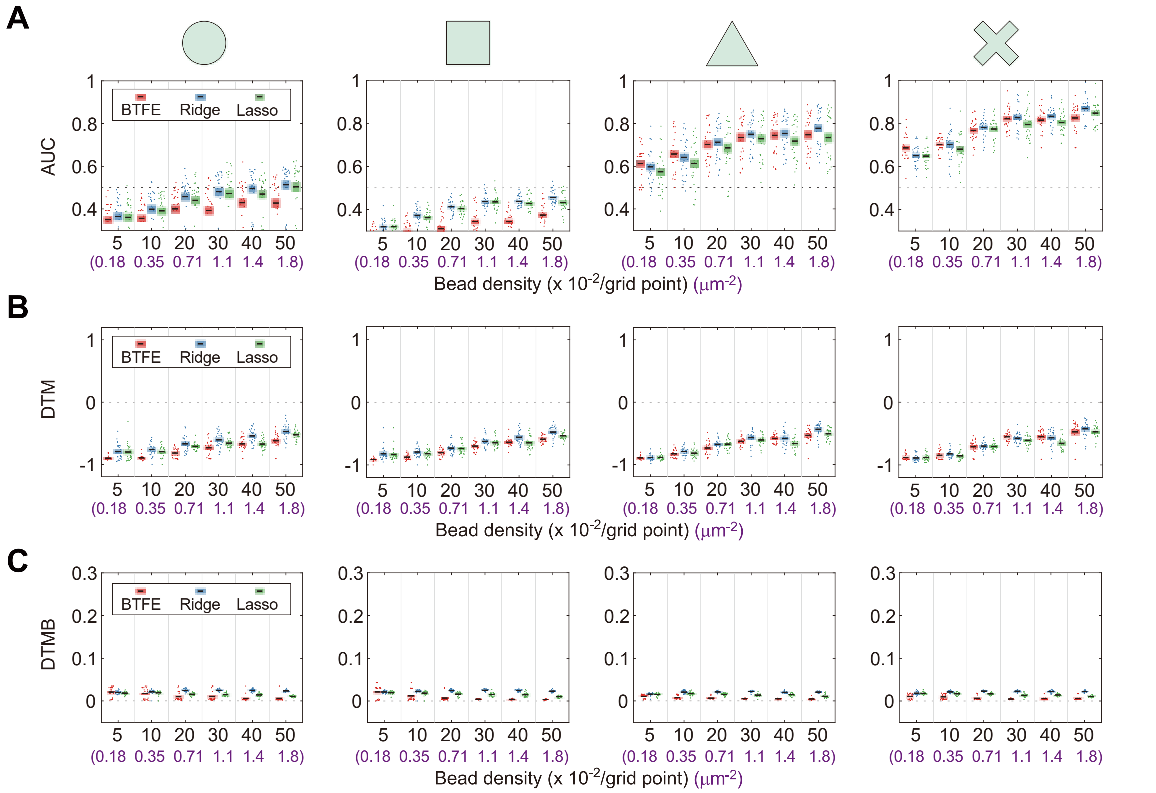

Figure S8. Relationship between geometric boundaries and force estimation accuracy in the absence of bead displacement noise.

From left to right, the force estimation accuracy is presented for cells with boundaries of a perfect circle, square, equilateral triangle, and X-shape, using noise-free bead displacement data. Following the format of **Figs. 5***D*–*F*, the force estimation is displayed in terms of (A) AUC, (B) DTM, and (C) DTMB.

Supplementary references

1. Colding, T.H., W.P. Minicozzi, and E.K. Pedersen. 2015. Mean curvature flow. *B Am Math Soc*. 52:297–333.

2. Mitchell, I.M. 2008. The flexible, extensible and efficient toolbox of level set methods. *J Sci Comput*. 35:300–329.
