# Supplementary material for: Bayesian traction force estimation using cell boundary-dependent force priors
Source: Biophys J. 2023 Nov 2;122(23):4542–54. doi: 10.1016/j.bpj.2023.10.032 (PMC10719052; doi:10.1016/j.bpj.2023.10.032)
Supplement: Document S2. Article plus supporting material [file mmc2.pdf]

Biophysical Journal
Article

Biophysical Society

Bayesian traction force estimation using cell boundary-dependent force priors

Ryosuke Fujikawa,[1] Chika Okimura,[2] Satoshi Kozawa,[1] Kazushi Ikeda,[1,3] Naoyuki Inagaki,[1] Yoshiaki Iwadate,[2] and Yuichi Sakumura[1,3,*]
[1]Graduate School of Science and Technology, Nara Institute of Science and Technology, Ikoma, Nara, Japan; [2]Department of Biology, Yamaguchi University, Yamaguchi, Japan; and [3]Data Science Center, Nara Institute of Science and Technology, Ikoma, Nara, Japan

ABSTRACT Understanding the principles of cell migration necessitates measurements of the forces generated by cells. In traction force microscopy (TFM), fluorescent beads are placed on a substrate's surface and the substrate strain caused by the cell traction force is observed as displacement of the beads. Mathematical analysis can estimate traction force from bead displacement. However, most algorithms estimate substrate stresses independently of cell boundary, which results in poor estimation accuracy in low-density bead environments. To achieve accurate force estimation at low density, we proposed a Bayesian traction force estimation (BTFE) algorithm that incorporates cell-boundary-dependent force as a prior. We evaluated the performance of the proposed algorithm using synthetic data generated with mathematical models of cells and TFM substrates. BTFE outperformed other methods, especially in low-density bead conditions. In addition, the BTFE algorithm provided a reasonable force estimation using TFM images from the experiment.

SIGNIFICANCE Estimating the force exerted by cells on an external substrate is important; however, previous studies have relied on estimating stress from substrate strain, which is not equivalent to cell traction force. Cell boundary is maintained by the internal stresses and forces from the substrate at the time and contains information about those forces. This study presents a novel approach using Bayesian statistics to estimate cell traction force by considering both substrate strain and cell boundary. Previous methods did not mathematically describe forces that depend on cell boundary; however, the present study successfully does so, leading to improved accuracy in estimating cell traction force.

INTRODUCTION

Cell motility is critical for biological functions, and understanding it aids in elucidating immune responses (1,2), neural network formation (3–6), and cancer invasion principles (7,8). Researchers have used various techniques such as elastic micropillar devices (9) and traction force microscopy (TFM) (10) to investigate how cells generate mechanical forces (11–14). Elastic micropillar devices consist of pillars arranged vertically in two dimensions, allowing researchers to estimate the force produced by a cell as the pillars bend when the cell moves at the pillars' tips. However, estimating accuracy is controversial because the two-dimensional (2D) arrangement of pillar tips differs from a continuous cellular substrate (9,15). By contrast, TFM measures substrate deformation by cell forces, where the deformation is observed as bead displacement on an elastic substrate's surface (Fig. 1 *A*). In previous studies, cell forces have been estimated by inverse calculations from bead displacement using weighted-sum and simultaneous linear equations of force (10,16). However, the observed bead displacements is typically fewer than the number of force points to be estimated, which makes solving simultaneous linear equations of force difficult. Experimental techniques and force-estimation algorithms have been developed to solve this issue; however, room exists for improving estimation accuracy.

An experimental improvement of force estimation involves measuring cytoskeletal fibers and focal adhesion (17,18). Large cells such as T3T fibroblasts generate strong forces within the range 0.25–0.5 kPa through mechanical links between integrins and substrates, which makes observations of cytoskeletal fibers and focal adhesions useful for force estimation. However, small motile cells such as nerve growth cones and immune cells generate force differently

Submitted May 4, 2023, and accepted for publication October 30, 2023.
*Correspondence: saku@bs.naist.jp
Editor: Anne Kenworthy.
https://doi.org/10.1016/j.bpj.2023.10.032
© 2023 Biophysical Society.
This is an open access article under the CC BY license (http://creativecommons.org/licenses/by/4.0/).

by exerting weak traction through clutch molecules via actin retrograde flow, mainly near the boundaries (11,19) (Fig. 1 *B*); neuronal growth cones generate a traction stress of ~10 Pa. For such cells, fast cycles of binding and detachment via weak adhesion through clutching are more practical for movement. Therefore, force estimation based on actin retrograde flow is suitable for small motile cells. Another experimental improvement is to increase bead density with two different fluorescent colors (17) or use high-resolution microscopy (20); however, high-density beads can result in fluorescence fusion and heterogeneous changes in physical properties of substrate due to inclusions in an elastic body (21–23). These observations suggest that a trade-off relationship exists between improved resolution and reduced quantitation. Even at high densities, the number of beads is relatively small when measuring forces in small cell regions such as neuronal growth cones. Thus, an algorithm for accurately estimating force at low bead densities is needed.

Various force-estimation algorithms have been used previously, including the boundary element method (10,24), Fourier transform traction cytometry (20,25–27), and the finite element method (28). All these algorithms have constraints when used to estimate forces. The ridge regression used in the boundary element method constrains the magnitude of the force squared and estimates small forces over a wide range (17,29,30) (Fig. 1 *C*). The lasso regression, which is used in the boundary element method and Fourier transform traction cytometry, selects fewer nonzero forces or fewer numbers of forces to account for bead displacements, estimating large forces within a narrow range (29) (Fig. 1 *D*). These algorithms assume that the forces occur around the bead's locations and are independent of cell boundary in most cases. A study that considers cell boundary would at least underestimate the forces because it would ignore bead displacements outside the cellular region (25). The cellular forces causing these displacements cannot be ignored because substrate deformations away from the cell, although small, are induced by significant intracellular forces (Fig. 1 *E*). TFM images show that cells with different boundaries should produce different forces to induce the same bead displacements, implying the importance of individual cell boundaries in force estimation. Although previous TFM studies have considered cell boundary, they have used common cell constraints of force points (31) and force direction (14,24); they have not considered individual cell boundaries.

Biologists have discovered that cell forces are greater near boundaries where actin polymerization is active (14,32,33) and are often directed inward (33–36). Inhibiting actin polymerization leads to a spherical cell boundary similar to a soap bubble (37) because it is energetically favorable. In the absence of substrate force, cells cannot spread (38) and the pseudopodia generate substantial passive retraction forces to make the cell spherical (39). Within a dense tissue of cells, many cells have similar shapes because of mechanical constraints (40). These biological findings can enhance force-estimation accuracy; however, incorporating them into mathematical models is difficult because of the complex and unique nature of cell boundaries. The inability to translate cell boundaries into mathematical force descriptions has been a major obstacle. However, developing cell-boundary-dependent constraints using mathematical formulas can promote force-estimation accuracy. Overcoming this bottleneck would represent a significant breakthrough in force estimation.

Here, we used prior knowledge of cell forces to estimate them on the basis of Bayesian statistics (specifically, Bayesian traction force estimation (BTFE)) (Fig. 1 *F*). The force magnitude prior was expressed as a decreasing function of distance from the cell boundary, whereas force direction priors were defined using the level-set method (LSM) and mean curvature flow (MCF) (41–43). This approach enabled us to mathematically define force direction at all locations within the cell. We computed the posterior distribution from prior knowledge of the forces and the likelihood of bead displacements and evaluated the accuracy of the estimated forces on synthetic data. BTFE was found to be more accurate than ridge regression and lasso regression. We also carried out force estimation using TFM images of a neuronal growth cone, a *Dictyostelium* cell, and a fish epidermal keratocyte and found that BTFE provided more reasonable force estimations than ridge regression and lasso regression.

METHODS

Traction force estimation with Bayesian framework

Likelihood of substrate deformation

Let $\mathbf{x} = (x, y)^T$ denote the substrate coordinates (where T denotes transposition). Suppose a mechanical force $\mathbf{f}(\mathbf{x}) = (f_x(\mathbf{x}), f_y(\mathbf{x}))^T$ applied at $\mathbf{x}$ deforms the substrate by $\mathbf{u}(\mathbf{x}) = (u_x(\mathbf{x}), u_y(\mathbf{x}))^T$. The deformation of each coordinate is determined by the sum of forces generated at all locations. If the substrate is isotropic and homogeneous, and $\mathbf{u}(\mathbf{x})$ is small, we can express $\mathbf{u}(\mathbf{x})$ as a spatial integral of $\mathbf{f}(\mathbf{x})$ (16),

$$\mathbf{u}(\mathbf{x}) = \int_\Omega \tilde{G}(\mathbf{x} - \mathbf{x}')\mathbf{f}(\mathbf{x}')d\mathbf{x}', \tag{1}$$

where Ω represents the entire substrate area and $\tilde{G}(\mathbf{x})$ represents the Boussinesq-Green function describing the elastic properties of the substrate,

$$\tilde{G}(\mathbf{x}) \equiv \frac{1-\nu}{\pi E r^3}\begin{pmatrix} (1-\nu)r^2 + \nu x^2 & \nu xy \\ \nu xy & (1-\nu)r^2 + \nu y^2 \end{pmatrix}. \tag{2}$$

Eq. 2 defines the range and degree of substrate deformation caused by forces, where ν and E are the Poisson's ratio and the Young's modulus of the substrate, respectively, and r represents the distance between the force point and a point on the substrate ($r = |\mathbf{x}|$). Our goal is to use a Bayesian

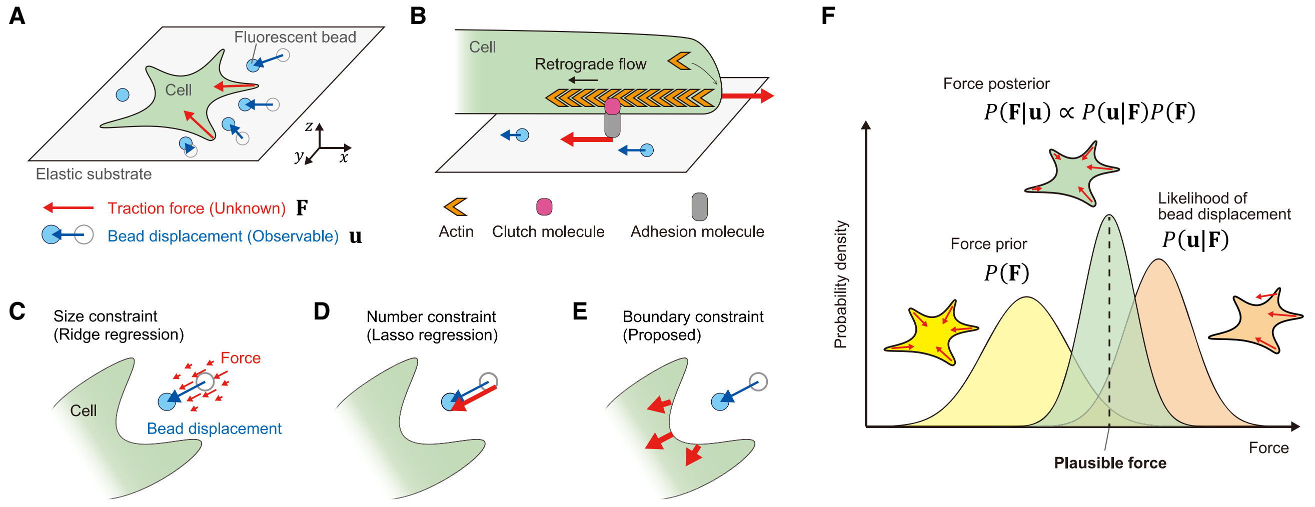

FIGURE 1 Diagram of Bayesian framework for estimating cell traction force using cell boundary priors. (*A*) Overview of traction force microscopy (TFM), a technique for measuring the force (**F**; *red arrows*) exerted by a cell (*green*) on a substrate. In TFM, fluorescent beads on the surface of the substrate (**u**; *blue circles and arrows*) move in response to the cell's traction force, allowing the force to be calculated through inverse analysis of the bead displacement. (*B*) Main source of cellular force, which is generated by the polymerization of actin molecules at the cell edge, forming actin filaments that push against the membrane. The reaction force of this polymerization causes the retrograde flow of actin filaments (*black arrow*) in an intracellular direction. The mechanical linkage between the actin filaments and the substrate, mediated by adhesion and clutch molecules, allows the retrograde flow to produce a traction force (*red arrow*) (*C*) Force estimation with magnitude constraints (ridge regression) reduces the force magnitude to avoid overfitting to the observed bead displacement. (*D*) Force estimation by number constraint (lasso regression) explains the bead displacement with only a small number of forces. (*E*) Force-estimation method proposed in the present study incorporates a cell boundary constraint, assuming that forces limited to the cellular region induce displacement of extracellular beads. (*F*) Conceptual diagram of Bayesian force estimation is shown, in which a posterior distribution ($P(\mathbf{F}|\mathbf{u})$; *green*) is obtained by satisfying the likelihood of the bead displacement **u** produced by the force $\boldsymbol{F}$ ($P(\mathbf{u}|\boldsymbol{F})$; *orange*) and the prior distribution of the force that the cell is likely to generate on the basis of its boundary ($P(\mathbf{F})$; *yellow*). The most plausible force is then estimated using MAP estimation.

approach to calculate the force $\mathbf{f}(\mathbf{x})$ on the basis of the displacement $\mathbf{u}(\mathbf{x})$. However, obtaining $\mathbf{f}(\mathbf{x})$ as a continuous function of $\mathbf{u}(\mathbf{x})$ is technically impossible. Thus, we discretized the substrate space using coordinates $\mathbf{x}'_n = (x'_n, y'_n)^T$ ($n = 1,\ldots,N$) to approximate the right-hand side of Eq. 1 by

$$\mathbf{u}(\mathbf{x}) \approx \frac{A}{N}\sum_{n=1}^{N}\tilde{G}(\mathbf{x} - \mathbf{x}'_n)\mathbf{f}(\mathbf{x}'_n), \quad (3)$$

where A denotes the area of the cellular region Ω, and A/N denotes the area of a single discretized area. Force estimation was conducted using a default 30×30 grid ($N = 900$; Fig. S1 *A*). Then, $(A/N)\mathbf{f}_0(\mathbf{x}'_n)$ represents the force per unit area at point $\mathbf{x}'_n$. Increasing the number of force-estimation points N on the substrate improves the spatial resolution of the force. However, a large value of N increases the number of unknown forces that need to be estimated; thus, N cannot be too large.

TFM utilizes the displacement of fluorescent beads placed randomly on the substrate to observe its partial deformation (as shown in Fig. 1 *A*). Let $\mathbf{u}_{bead} = (u_x(\mathbf{x}_1), u_y(\mathbf{x}_1), \ldots, u_x(\mathbf{x}_B), u_y(\mathbf{x}_B))^T$ denote the true displacement of B beads, where $\mathbf{x}_i = (x_i, y_i)^T$ ($i = 1,\ldots,B$) represents the coordinates of the i-th bead. We can express this displacement using Eq. 3:

$$\mathbf{u}_{bead} = G\mathbf{f}$$

$$\mathbf{f} = \left(f_x(\mathbf{x}'_1), f_y(\mathbf{x}'_1), \ldots, f_x(\mathbf{x}'_N), f_y(\mathbf{x}'_N)\right)^T \times \frac{A}{N}$$

$$G = \begin{pmatrix} \tilde{G}(\mathbf{x}_1 - \mathbf{x}'_1) & \cdots & \tilde{G}(\mathbf{x}_1 - \mathbf{x}'_N) \\ \vdots & \ddots & \vdots \\ \tilde{G}(\mathbf{x}_B - \mathbf{x}'_1) & \cdots & \tilde{G}(\mathbf{x}_B - \mathbf{x}'_N) \end{pmatrix}.$$

Given the Gaussian observation noise, we can describe the measured bead displacement $\mathbf{u}_{obs}$ using a probabilistic model (likelihood) whose mean is the true displacement $\mathbf{u}_{bead} = G\mathbf{f}$,

$$P(\mathbf{u}_{obs}|\mathbf{f}) = N\left(\mathbf{u}|G\mathbf{f}, \alpha^{-1}I_{2B}\right), \quad (4)$$

where I_{2B} is the $2B$-dimensional unit matrix and α^{-1} is the precision parameter (inverse of the variance). We assumed that the x and y components of the variance were independent and set $\alpha = 1$.

Traction force prior

We assumed that the traction force's prior distribution was a 2D Gaussian distribution, with the center expressed as a product of the magnitude and direction that depended on cell boundary. In the first step, we defined the magnitude and direction using a common algorithm for the entire cell area. In the second step, we introduced local-dependent variations in the magnitude and direction and estimated them. The prior distribution of forces biases the estimation rather than limiting it to a specific range. Thus, the possibility remains that bead displacement will cancel the effect of the prior distribution.

Step 1: Design for whole-cell region-dependent magnitude and direction. The traction force is larger, closer to the cell boundary, and oriented toward the cell interior (Fig. 2 *A*). We described the traction force center $\mathbf{f}_\mu(\mathbf{x}'_n)$ as the product of the magnitude $m(\mathbf{x}'_n)$ and the unit vector $\mathbf{d}(\mathbf{x}'_n)$:

$$\mathbf{f}_\mu(\mathbf{x}'_n) = m(\mathbf{x}'_n)\mathbf{d}(\mathbf{x}'_n). \quad (5)$$

We modeled the magnitude as a decay function of the inward cell distance $r(\mathbf{x})$ from the boundary of each coordinate (Fig. 2 *B*),

$$m(r) = \exp\left(-r^2/\sigma^2\right), \quad (6)$$

where we set the hyperparameter σ^2 to 10% of the cell area; larger cells generate a force over a wider area from the boundaries. For example, the

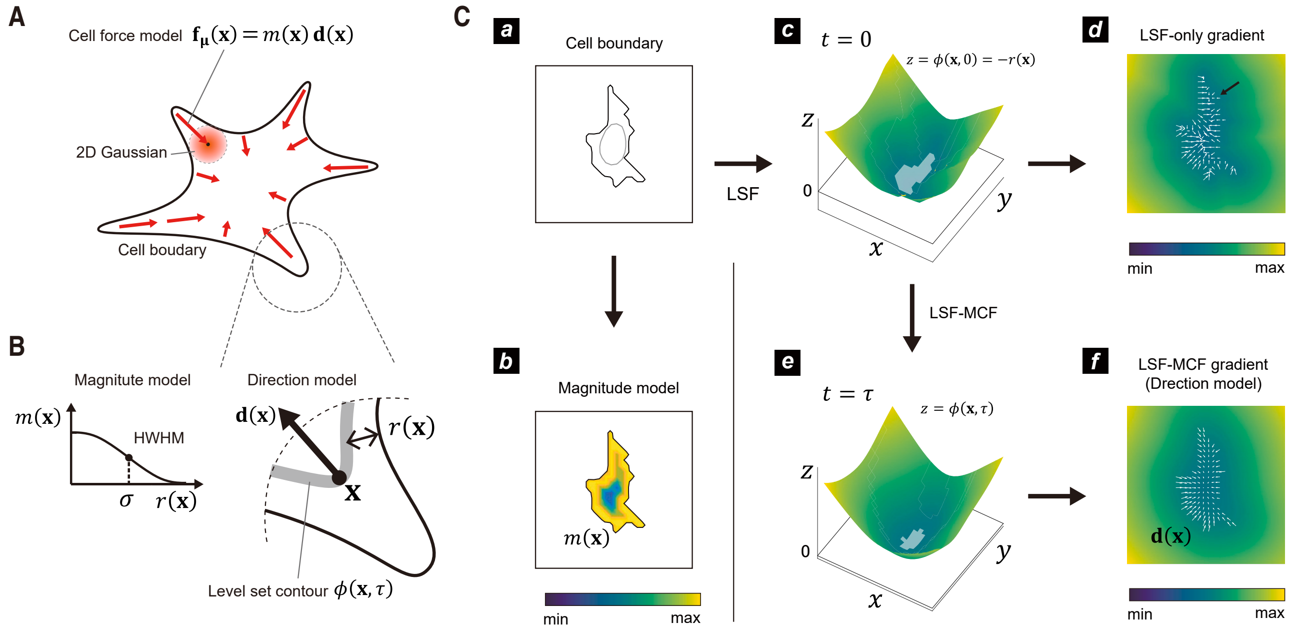

FIGURE 2 Model of force prior derived from cell boundary. (*A*) A model of inward cellular traction force and its components. The prior probability distribution of the traction force at position $\mathbf{x}$, $P(\mathbf{f}(\mathbf{x}))$, was defined as a 2D Gaussian distribution centered on the mean force $\mathbf{f}_\mu(x)$, which is divided into magnitude $m(\mathbf{x})$ and direction $\mathbf{d}(\mathbf{x})$. (*B*) Models for the magnitude $m(\mathbf{x})$ and direction $\mathbf{d}(\mathbf{x})$ functions. The magnitude function $m(\mathbf{x})$ was set to be a nonnegative Gaussian function that depends on the distance $r(\mathbf{x})$ from the nearest-neighbor edge. For the force direction function $\mathbf{d}(\mathbf{x})$, the cell boundary was represented as a level-set function (LSF), and the unit vector of the gradient of the LSF was introduced. (*C*) Calculation of force magnitude and direction based on cell morphology. (*a*) Boundary of the cultured cell (*solid line*) compared to the boundary transformed using the LSF with MCF (see *e*). (*b*) Force magnitude $m(\mathbf{x})$ calculated and visualized with a color gradient. (*c*) Cell boundary represented by the LSF $z = \phi(\mathbf{x},0)$, with the gray region denoting the original cell boundary $\phi(\mathbf{x},0) = 0$. The z axis represents the distance from the cell edge: $-r(\mathbf{x})$ for inside and $r(\mathbf{x})$ for outside. (*d*) Gradient of the LSF inside the original cell boundary, with white arrows representing unit vectors. Regions with biologically inappropriate directions are indicated by the black arrow. (*e*) LSF after the cell has been transformed by time τ with MCF, $\phi(\mathbf{x},\tau)$, where the time of the original cell boundary is zero. The gray region represents the interior of the transformed cell boundary $\phi(\mathbf{x},\tau) = 0$, with the edges represented by dotted circles in (*a*). (*f*) Gradient of the transformed boundary in (*e*), with white arrows indicating unit vectors of gradients inside the original cells, defined as $\mathbf{d}(\mathbf{x})$.

model of the magnitude of the traction force for the HT1080 cell boundary (Fig. 2 *C–a*) is shown in Fig. 2 *C–b*.

We used the LSM (44,45) to define the force direction $\mathbf{d}(\mathbf{x}'_n)$ as a boundary-dependent inward direction. LSM represents the 2D cell boundary in 3D space using the distance $r(\mathbf{x})$ from each $\mathbf{x}$ to the nearest-neighbor cell boundary as the z coordinate. The boundary of the HT1080 cells (Fig. 2 *C–a*) was transformed into the 3D surface shown in Fig. 2 *C–c*, with the level-set function (LSF), $z = \phi(\mathbf{x}, t)$, representing the entire surface. The coordinate set $\{\mathbf{x}\}$ satisfying $\phi(\mathbf{x}, t = 0) = 0$ gives the 2D cell boundary at time $t = 0$, with the negative region of the function $z = \phi(\mathbf{x}, t = 0)$ indicating the cell interior and the gradient of this region denoted as $\mathbf{d}(\mathbf{x}'_n)$. However, the LSF gradients in the complex cell boundary are complicated and lack continuity (Fig. 2 *C–d*) because gradients near boundaries have perpendicular orientations, which generate gradients in opposite directions close together, especially in thin pseudopodia. In addition, a thin structure such as a pseudopod generates forces parallel to its direction (Fig. 1 *A*). Therefore, the LSF gradient of the original cell boundary is not suitable for the center of the traction force prior.

We incorporated the effect of the energy consumption required for cells to build and maintain complex boundaries into our force direction model. Cells resist forces that push them toward a spherical boundary by expending mechanical energy. This force is generated by the cells. In our simulation, we used the MCF algorithm to transform the cell boundary toward a spherical boundary for a brief time (τ). We then used the gradient of the LSF as the force direction, which was obtained after the transformation (supporting methods). The boundary of the simulation ($\phi(\mathbf{x}, \tau) = 0$) showed a smooth boundary with no protrusions (dotted lines in Fig. 2 *C–a* and *C–e*). To obtain a smooth gradient distribution, we cut out the original cellular region from the gradient distribution of the transformed 3D surface $z = \phi(\mathbf{x}, \tau)$ (Fig. 2 *C–f*), which we defined as the model of the force direction ($\mathbf{d}(\mathbf{x}'_n)$).

Step 2: Introduction of locality dependence. To correct for variations in direction and magnitude of forces due to unobservable local conditions in cells, we introduced a correction component in $\mathbf{f}_\mu(\mathbf{x}'_n)$. We defined the hyperparameters $\{\theta_n, s_n\}$ ($n = 1, \cdots N$) as the correction parameters for force direction and magnitude at coordinate $\mathbf{x}'_n$ and transformed $\mathbf{f}_\mu(\mathbf{x}'_n)$ to

$$\mathbf{f}_n\left(\mathbf{x}'_n, \theta_n, s_n\right) \equiv s_n \begin{pmatrix} \cos\theta_n & -\sin\theta_n \\ \sin\theta_n & \cos\theta_n \end{pmatrix} \mathbf{f}_\mu\left(\mathbf{x}'_n\right). \tag{7}$$

A conceptual diagram illustrating the relationship between $\mathbf{f}_\mu$ and $\mathbf{f}_n$ and the prior design characterized by hyperparameters are provided in Fig. S1 *B*. The set of force centers $\mathbf{F}_\mu\{\theta_n, s_n\}_{n=1}^{N} = (\mathbf{f}_1(\mathbf{x}'_1, \theta_1, s_1)^T, \cdots, \mathbf{f}_N(\mathbf{x}'_N, \theta_N, s_N)^T)^T$ was used to define the prior of the cell forces as a 2D Gaussian:

$$P\left(\mathbf{f} \middle| \{\theta_n, s_n\}_{n=1}^{N}\right) = N\left(\mathbf{f} \middle| \mathbf{F}_\mu\{\theta_n, s_n\}_{n=1}^{N}, \beta^{-1} I_{2N}\right), \tag{8}$$

where $\beta = 0.1$ is the variance of the Gaussian and I_{2N} is a $2N$-dimensional unit matrix. Because the angle and magnitude distributions of forces are likely to be independent, we defined the prior probability distribution $P(\{\theta_n, s_n\})$ for $\{\theta_n, s_n\}$ as $P(\{\theta_n, s_n\}) = P(\{\theta_n\})P(\{s_n\})$, where $P(\{\theta_n\})$ is uninformative (i.e., constant value independent of $\{\theta_n\}$). For the prior of $\{s_n\}$, we introduced the Laplace distribution,

$$P\left(\{s_n\}_{n=1}^{N}\right) = \exp\left\{-\gamma\sum_{n=1}^{N}|s_n|\right\}, \tag{9}$$

where $\gamma = 0.01$ is the reciprocal scale parameter. The Laplace prior has the effect of reducing the size of as many forces as possible to zero, which is the same as that of the lasso regression.

Traction force posterior

The traction force posterior, according to the Bayesian theorem, is proportional to the likelihood (Eq. 4) multiplied by the prior (Eq. 8) (Fig. 1 *G*), expressed as

$$P\left(\mathbf{f}\middle|\mathbf{u}_{obs},\{\theta_n,s_n\}_{n=1}^{N}\right) \propto P(\mathbf{u}_{obs}|\mathbf{f}) \times P\left(\mathbf{f}\middle|\{\theta_n,s_n\}_{n=1}^{N}\right). \tag{10}$$

The force that satisfies both the bead displacement (likelihood) and cell boundary constraints (prior) and maximizes the posterior probability density (maximum a posteriori (MAP)) can be determined. We obtain the MAP estimation by taking the logarithm of this equation, which is

$$\begin{aligned}\mathbf{f}_{est} &= \underset{\mathbf{f}}{\operatorname{argmax}}\left\{\log P\left(\mathbf{f}\middle|\mathbf{u}_{obs},\{\theta_n,s_n\}_{n=1}^{N}\right)\right\}\\ &= \underset{\mathbf{f}}{\operatorname{argmax}}\left\{-\frac{\alpha}{2}|\mathbf{u}_{obs} - G\mathbf{f}|^2\right.\\ &\quad\left. -\frac{\beta}{2}\left|\mathbf{f} - \mathbf{f}_{\mu}\{\theta_n,s_n\}_{n=1}^{N}\right|^2\right\},\end{aligned} \tag{11}$$

where α, β, and $\{\theta_n, s_n\}$ $(n= 1,\cdots N)$ are hyperparameters. Using the expectation-maximization (EM) algorithm, we calculated approximate solutions for the hyperparameters $\{\theta_n, s_n\}$ $(n= 1,\cdots N)$ and optimal force (supporting methods). A conceptual diagram illustrating the EM algorithm is provided in Fig. S1 *B*.

In the context of Bayesian estimation, hyperparameters define the properties of the prior distribution, serving to balance the likelihood and prior distribution; an example is the regularization parameter in ridge or lasso regression. A large value of this parameter suppresses the size of the regression coefficients or the number of nonzero regression coefficients to prevent overfitting the model. In this study, we introduced four hyperparameters independent of individual cells. We set the magnitude of the observed noise in the bead position as $\alpha= 1$ (Eq. 4) and the constraint level of the estimated force vector as $\beta= 0.1$ (Eq. 8), representing the weights of the likelihood and prior distributions, respectively. In simple terms, the optimization assigns approximately 10% of the weight to the prior distribution in comparison to the likelihood (see Eq. 11). The force magnitude constraint level, based on the entire cell area, is expressed as $\sigma^2 = 0.1\times$ area of the cell region (Eq. 6), varying with cell size. The local size constraint level is set to $\gamma= 0.01$ (Eq. 9), limiting the number of nonzero forces to prevent excess. In contrast, a set of hyperparameters, $\{\theta_n, s_n\}$ $(n = 1,\cdots N)$, represent unobservable hidden factors, estimated simultaneously with the forces (see supporting methods). Those are depending on individual cell boundaries, and specific values are not provided here to avoid complication. Adopting a Bayesian statistical approach allows for effective modeling and more accurate estimation of such hidden factors.

Synthetic data of cell force and bead displacements

Evaluating the algorithm's force-estimation accuracy requires a comparison to the correct force. Because the exact force of a living cell is unknown, we generated synthetic datasets of model cell traction and bead displacements using mathematical models of cells and bead-embedded substrates (Fig. 3 *A*). The placements of traction forces and beads were independent of each other, and we used displaced beads to estimate traction forces and assess the difference from the true forces generated by the model cell. We used edge boundaries of HT1080 cells to introduce them into model cells (Fig. 3 *A–a*), generated 20 noisy traction forces inside the model cell (Fig. 3 *A–b*), randomly placed low-density beads on the model substrate (Fig. 3 *A–c*), calculated the bead displacement due to the traction force of the model cell (Eq. 3) (Fig. 3 *A–d*), and added observation noise to the bead displacement (Fig. 3 *A–e*). We used five different realistic cell boundaries (Fig. 3 *A–a* and *B*) and four geometric boundaries (Figs. S7 and S8) to perform force estimation using our proposed method, BTFE. For comparison, we also estimated forces using ridge and lasso regressions, whose regularization parameters were optimized by cross-validation. We did not perform fast Fourier transfer traction cytometry in this study because it requires dense beads. Because biological experiments can simultaneously observe cell boundary, we assumed that the boundary of the model cells in the present study could also be observed; the BTFE introduced them as prior knowledge.

Performance evaluation index

We evaluated the estimation accuracy using three indices: the area under the ROC curve (AUC), the deviation of traction magnitude (DTM) (17), and the deviation of traction magnitude in the background (DTMB) (46). We did not use mean-square error because it is not appropriate for comparing force distributions (47). To calculate the AUC, we considered the grid points at which the model cell generated traction as positive points and all other points as negative points. To determine the false-positive (FP) and true-positive (TP) rates, we introduced a threshold for the force magnitude estimated by the algorithm, which converted the analog value of force into a binary discrimination of positive or negative. A higher threshold results in only large forces being positive, whereas a lower threshold leads to more FPs. The AUC is higher for a smaller threshold with low FP and high TP rates (refer to Fig. S2 for details). The DTM is a metric that measures the accuracy of the estimated force by comparing it to the true force at positive points. It is defined as the average of the fraction of magnitude error between the estimated force $\mathbf{f}_{est}(\mathbf{x}_i^p)$ and the true force $\mathbf{F}(\mathbf{x}_i^p)$ at M_p positive points $(\mathbf{x}_i^p; i= 1,\cdots,M_p)$,

$$DTM = \frac{1}{M_p}\sum_{i=1}^{M_p}\frac{|\mathbf{f}_{est}(\mathbf{x}_i^p)| - |\mathbf{F}(\mathbf{x}_i^p)|}{|\mathbf{F}(\mathbf{x}_i^p)|}. \tag{12}$$

The DTM approaches zero with improved accuracy of the estimated force magnitude, taking a positive value when the estimated force is greater than the true force and a negative value when it is less. DTMB, the third index, evaluates the noise level of the FP estimated force by comparing it to the average true force magnitude. DTMB is defined as the average ratio between the estimated force magnitude $|\mathbf{f}_{est}(\mathbf{x}_i^n)|$ and the average true force magnitude $E\langle\mathbf{F}(\mathbf{x}_i^p)\rangle$ at M_n negative points $(\mathbf{x}_j^n; j= 1,\cdots,M_n)$:

$$DTMB = \frac{1}{M_n}\sum_{j=1}^{M_n}\frac{\left|\mathbf{f}_{est}\left(\mathbf{x}_j^n\right)\right|}{E\langle\mathbf{F}(\mathbf{x}_i^p)\rangle}. \tag{13}$$

The DTMB takes values greater than zero and approaches a value of 1 when the FP force is closer to the average of the true force. If DTMB is a small value close to zero, then the FPs do not have significant magnitudes.

Experimental procedure for bead displacement measurement

Neurons were cultured on polyacrylamide gel substrates as previously described (11,48,49). Glass-bottom dishes were treated with 0.1 N NaOH for 15 min and then with 2% (v/v) 3-aminopropyltrimethyoxysilane (Sigma-Aldrich) in 2-propanol for 15 min. After the dishes were washed with H_2O, 0.5% glutaraldehyde (Sigma-Aldrich) solution was applied for 30 min. The dishes were then washed with H_2O and dried. Acrylamide

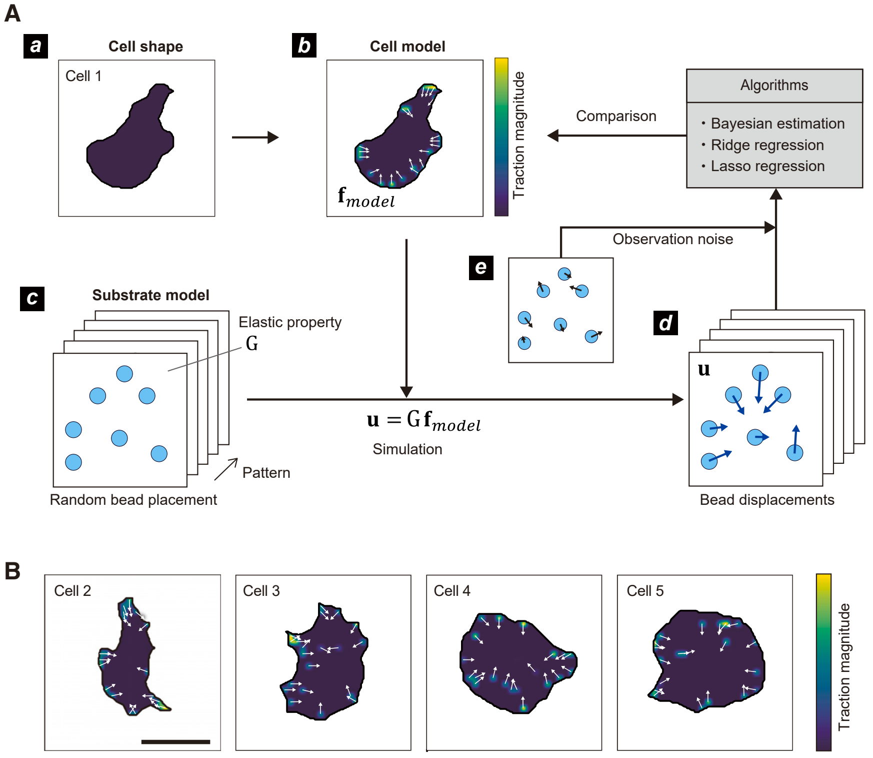

FIGURE 3 Procedure for generating synthetic bead displacements and force estimation. (*A*) Workflow used to estimate traction forces from a synthetic dataset. First, we (*a*) extracted the model cell boundaries from cultured cells and (*b*) generated a virtual traction force inside it. The direction and magnitude of the forces are indicated by white arrows and the background color, respectively. Next, we (*c*) randomly distributed beads on the model substrate and (*d*) calculated their displacement due to traction force using the Boussinesq approximation ($\mathbf{u} = \mathrm{G}\mathbf{f}$). (*e*) The synthetic data for bead displacement were obtained by adding observation noise (2D Gaussian distribution with a standard deviation of 0.1 μm) to the displacement data. We used these noisy bead displacements to perform a Bayes estimation, ridge regression, and lasso traction force estimation and compared the estimated results to the correct cell forces to evaluate accuracy. (*B*) Cell models used for the synthetic dataset. We used five different cell boundaries and prepared different traction force distributions for each cell. For each cell boundary and traction force distribution pair, we prepared five different bead distributions.

and bis-acrylamide stock solutions (Nacalai tesque) were diluted to 3.75% and 0.03%, respectively, as previously reported (49). The Young's modulus was 0.269 $\pm$ 0.0242 kPa (mean $\pm$ SE, $n = 7$).

For *Dictyostelium* cells and fish epidermal keratocytes, bead displacement measurements were performed according to previously reported methods (50,51). The elastic substrates were made from a mixture of polydimethylsiloxane (CY-52-276 A and B, Dow Corning Toray, Tokyo, Japan) with a 10:8 ratio for *Dictyostelium* cells and a 6:10 ratio for keratocytes. The Young's moduli were estimated as 281 and 13.8 kPa, respectively.

RESULTS

BTFE is robust to bead location and density

We analyzed the force-estimation algorithms and evaluated their characteristics. BTFE, ridge, and lasso regression algorithms were used to estimate forces on the same traction forces of the model cells (Fig. 4 *A*) and two model substrates with different bead densities (Fig. 4 *B*). We compared the estimations made with low-density beads (Fig. 4 *C–E*) and middle-density beads (Fig. 4 *F–H*) for each algorithm. BTFE estimated forces only on the intracellular side, and the distribution was similar to the correct forces (Fig. 4 *C* and *F*). Ridge regression estimated small forces as a broad force field across cell boundaries because it constrains forces to be small (Fig. 4 *D* and *G*). Lasso regression, however, estimated larger forces than ridge regression and provided a stronger contrast between the presence and absence of forces because it constrains the forces to zero at many locations inside and outside the cell (Fig. 4 *E* and *H*). The ridge and lasso regression methods estimated numerous small forces outside the cell because of the observation noise in bead displacement of the substrate model. The ridge and lasso regressions overestimate on substrates that are not in contact with the cell because cells cannot exert force on a substrate with which they are not in contact.

In TFM, the beads' positions cannot be controlled; their influence must therefore be minimized. To test this approach, we performed force estimation of the same force distribution (Fig. S3 *A*) using low-density beads at two different locations (Fig. S3 *B*). We compared the results obtained using beads with location pattern X (Fig. S3 *C–E*) with those obtained using beads with location pattern Y (Fig. S3 *F–H*) for each algorithm. We found that the ridge and lasso regressions produced substantially different estimates depending on the bead location, whereas the BTFE estimates were relatively stable. We obtained similar results when validating with medium bead densities (Fig. S4). The ridge and lasso regressions estimate forces around the bead location with constraints on the magnitude or number of forces, making the estimation results sensitive to bead location. By contrast, Bayes force estimation constrains the location, direction, and magnitude of forces on the basis of cell boundary, making it more robust to differences in bead locations. To accurately evaluate force estimations, it is necessary to conduct estimates using various cell boundaries, bead distributions, and force distributions and to statistically verify the accuracy.

We verified that our algorithm exhibits low sensitivity to varying conditions. First, the EM algorithm used in this study updates estimates and is generally sensitive to the

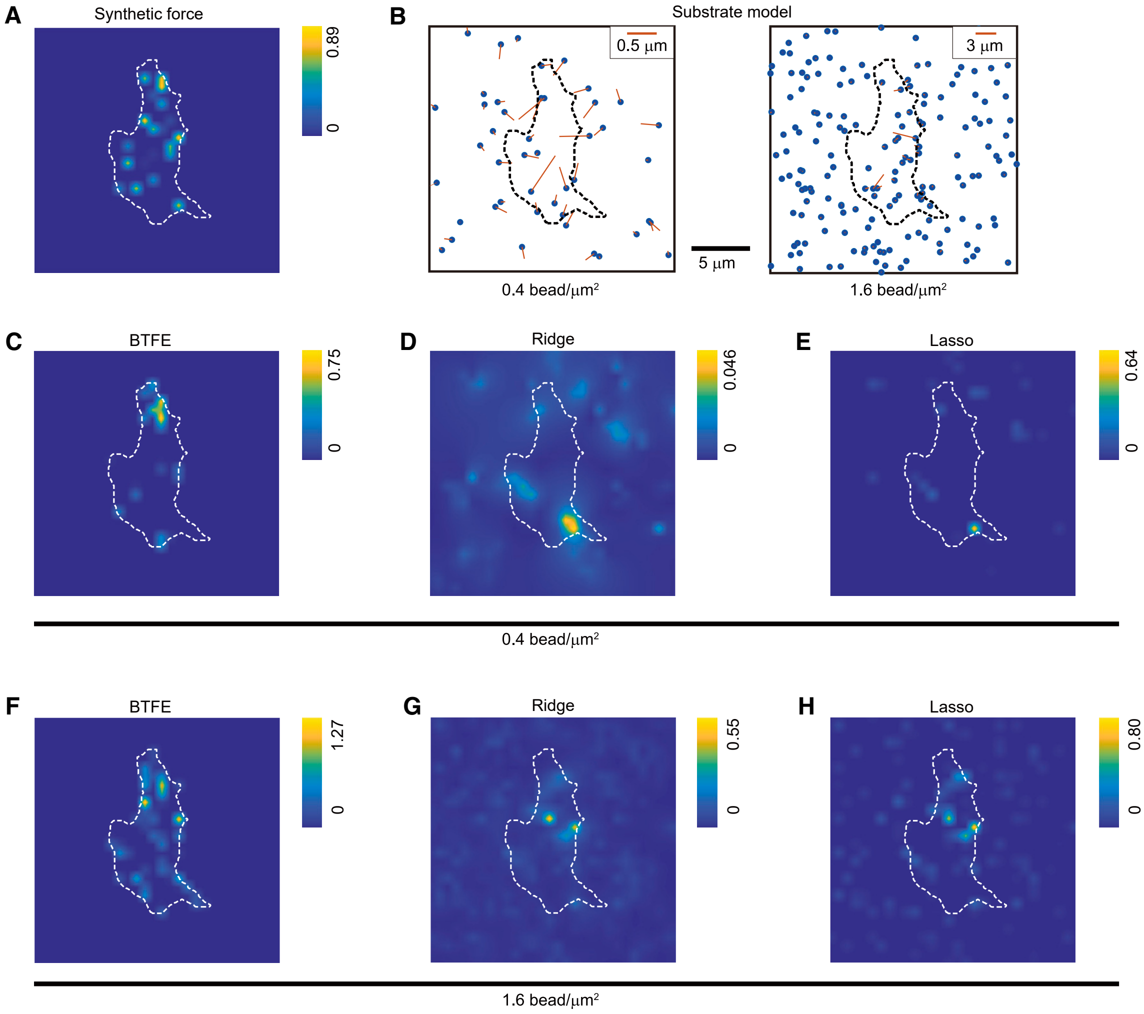

FIGURE 4 Variation in force-estimation results with different bead densities and distributions. (*A*) Examples of introduced synthetic forces, with the force magnitude indicated by the background color. There are 20 individual force points, each with inwardly directed force. (*B*) Randomly scattered beads on the substrate model (density: 0.4 and 1.6 bead/μm^2). The red lines indicate the bead displacements caused by the synthetic force shown in (A). Scale bar, 5 μm. (*C–E*) Comparison of estimation results from three different algorithms—(*C*) Bayes, (D) ridge, and (E) lasso—for a substrate model with a bead density of 0.4 bead/μm^2. All estimations were performed using the bead displacements shown in (*B*). (*F–H*) Same as (*C*)–(*E*) but with the substrate model having a bead density of 1.6 beads/μm^2. For all calculations, we placed estimated force points on a 30 $\times$ 30 grid.

initial values of these estimates. To address this issue, we conducted estimations starting from multiple different initial values, selecting the one that maximized the MAP density of the force. Next, we investigated the algorithm's sensitivity to the density of the grid used for force-estimation points (Fig. S5). We found that the BTFE estimates remained largely consistent irrespective of whether we used a denser grid (60 $\times$ 60). By contrast, ridge and lasso regression methods showed greater variation in their estimates. These results suggest that our estimation algorithm is robust to varying conditions.

BTFE improves estimation accuracy

The accuracy of algorithms was statistically compared using the AUC, DTM, and DTMB indices. The AUC was calculated by classifying estimated forces into four types: TP, false negative (FN), FP, and true negative (TN). TP occurs when a force is generated on the model substrate and the estimation result also has a force, FN occurs when a force is generated on the model substrate and the estimation result does not have a force, FP occurs when no force is generated on the model substrate and the estimation result has a force, and TN occurs when no force is generated on the model substrate and the estimation result does not have a force (Fig. 5 *A*; see Fig. S2 for details). DTM indicates how close the TP force's magnitude was to the model cell's true magnitude (Fig. 5 *B*), and DTMB represents the magnitude of the FP force relative to the average of the true force magnitudes (Fig. 5 *C*). Ten different bead position patterns were introduced for each of the five cell boundaries in Fig. 3 against a single force pattern, and the three indices were calculated

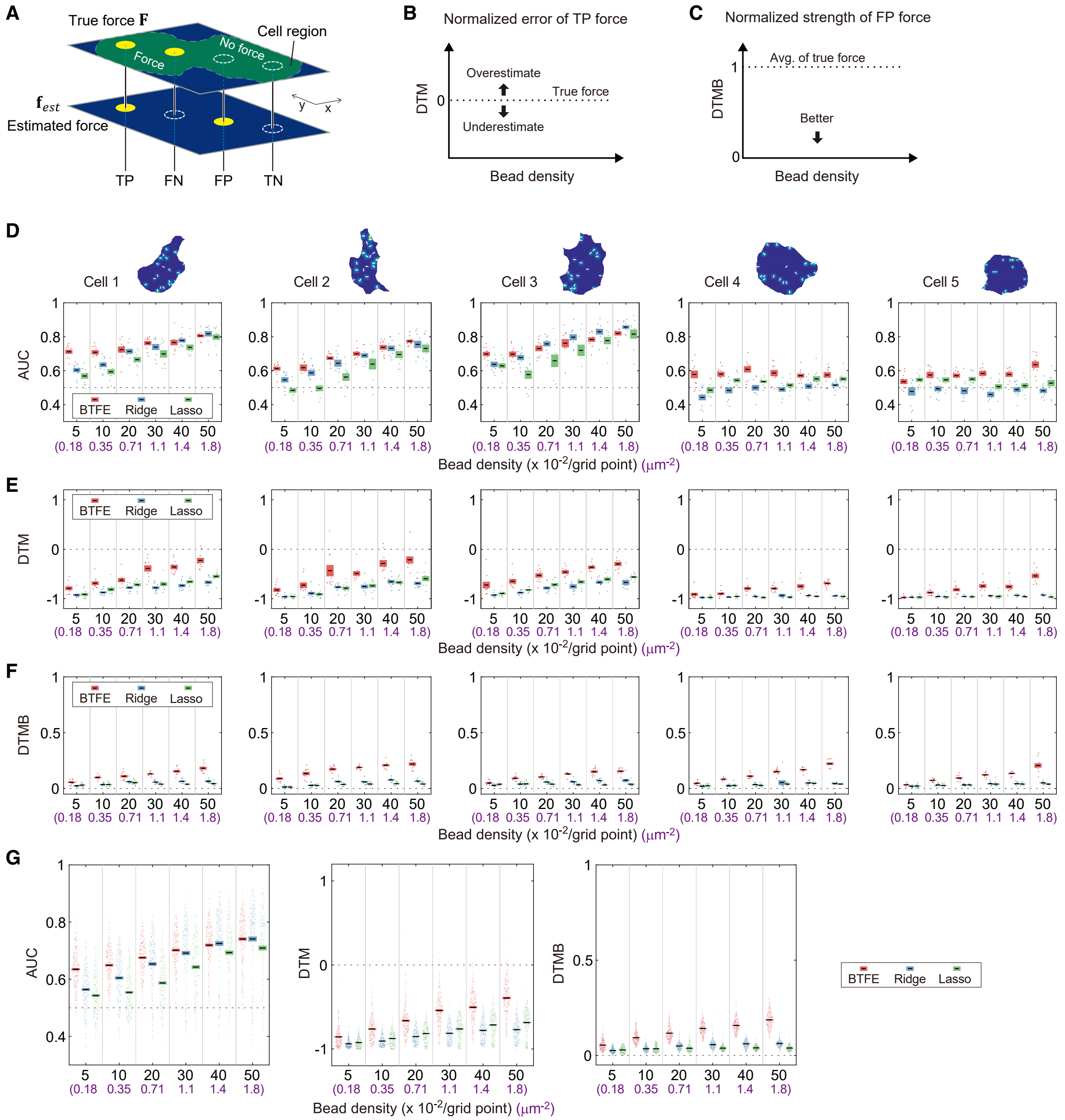

FIGURE 5 Performance comparison of traction force-estimation algorithms. (*A*) A diagram showing force detection classifications and evaluation measures. Four types of force detection were used: true positive (TP), false negative (FN), false positive (FP), and true negative (TN), which were used to create an ROC curve for force detection accuracy. See Fig. S2 for details on how the area under the ROC curve (AUC) was calculated. Only forces estimated in the cellular region were targeted for any estimation algorithms. (*B*) Deviation of traction magnitude (DTM) is the ratio of the difference between the estimated TP force and the actual force magnitude. The closer to zero, the better. A value of -1 indicates that the estimated force is very close to zero. (*C*) Deviation of traction magnitude in the background (DTMB) indicates how large the estimated FP force was relative to the average magnitude of the actual force. The closer to zero, the better. (*D*) AUC versus bead density for the five cell boundaries (Fig. 3). Bead density is shown as the number of beads per grid point and the number of beads per unit area (*purple*). One force distribution was introduced in each cell boundary (*top row*), and evaluations were done for 10 different patterns of bead positions (*bottom row*). Cells 1–3 have relatively complex boundaries, whereas cells 4 and 5 are relatively round. (*E*) Same as (*D*) but for comparisons of DTM versus bead density. (*F*) Same as (D) but for comparisons of DTMB versus bead density. (*G*) The three evaluations for all data (n = 250; five different cell boundaries, five different force generation patterns, 10 different initial bead placements). The error bars in (*D*)–(*G*) represent the SE. For all calculations, we placed estimated force points on a 30 × 30 grid.

(Fig. 5 *D–F*). We assessed the accuracy of force estimation within the cellular domain for all estimation algorithms, despite ridge and lasso regressions estimating forces beyond the domain.

In the AUC comparison (Fig. 5 *D*), the advantage of the Bayes approach was particularly significant at low bead densities; in addition, the performance of all the algorithms improved with increasing bead density because more information related to substrate deformation improved the accuracy of the force estimations. All the algorithms performed better when the cell boundary was complex (cells 1–3). Lasso regression showed that the AUC was ~0.5 for low-density beads, which is not different from random binary discrimination. When the cell had a nearly round boundary (cells 4 and 5), the ridge and lasso regressions' accuracy was ~0.5 in AUC at all densities, whereas that of the Bayes approach was ~0.6. The performance of all the methods was likely lower when the force distribution of the near-round cells was relatively isotropic. The forces that lead to isotropic bead displacement are also isotropic and lead to isotropic bead displacement even if the location of the force is shifted in the rotational direction.

We used DTM to examine how accurately the force estimated as a TP matched that of the model cell (Fig. 5 *E*). The DTM results reveal that, although all the estimation algorithms underestimated forces, the Bayes approach had a weaker effect on reducing estimates compared with the other methods. When the cell boundary is complex and the bead density is low or if the cell boundary is round, ridge or lasso regression-based estimation results in a DTM of approximately -1, indicating that the force estimation is very low. We also used DTMB to analyze the magnitude of the FP force relative to the average force of the model cells (Fig. 5 *F*). Among the three approaches, the Bayes approach had the highest FP forces, but the maximum ratio between FPs by BTFE and the average of true forces (DTMB = 1) was 0.3 at maximum, which is sufficiently small to be considered noise. Lastly, we compared the three indices using five different force distributions (250 combinations of cell boundaries and distributions of forces and beads) (Fig. 5 *G*). The results were similar to those obtained from a single force distribution (Fig. 5 *D–F*). These results suggest that the BTFE performs better than the other two indices, particularly in low-density bead conditions. These three evaluation metrics prompt the question: how impactful is the local force prior, a characteristic feature of BTFE? To address this question, we used five real cell boundaries to assess the accuracy when the prior is not adjusted locally. When we fixed the hyperparameters s and θ and carried out force estimation, we observed a decrease in the estimation accuracy (Fig. S6). This result underscores the importance of locally adjusting the prior.

In addition, to investigate the relationship between boundary symmetry and estimation accuracy, we used cell models with geometric boundaries—specifically, a perfect circle, square, equilateral triangle, and X boundary—to evaluate force-estimation accuracy. The perfect circle is entirely symmetrical, followed by increasing levels of asymmetry in the remaining boundaries. The substrate model was consistent with that shown in Fig. 3. Our computational results unambiguously revealed a trend where boundaries with greater symmetry yielded poorer AUC values (Fig. S7 *A*). The most complex X boundary provided the highest estimation accuracy. In comparisons among algorithms, BTFE excelled in accuracy with asymmetric boundaries such as the triangle and an X boundary with a low bead density but performed poorly with symmetrical boundaries. For DTM and DTMB, the performance differences were negligible (Fig. S7 *B* and C). Because geometric boundaries lack the boundary noise found in real cells, they are ideal for studying the effects of noise in bead displacement measurements. Accordingly, we conducted calculations similar to those represented in Fig. S6 but without including bead displacement noise. The accuracy of both the ridge and lasso regressions improved, whereas the accuracy of the BTFE remained largely unchanged (Fig. S8 *A*). Even under these conditions, the performance difference between DTM and DTMB remained minimal, although the ridge regression's DTM showed notable improvement (Fig. S8 *B* and *C*). These findings suggest that BTFE is robust against measurement noise in bead displacement.

BTFE estimates acceptable traction force from TFM images

We applied the force-estimation algorithms to TFM images of three motile cell types—specifically, neuronal growth cone, *Dictyostelium* cell, and fish epidermal keratocyte—with bead densities of 0.42, 3.34, and 0.38 beads/μm^2, respectively (Fig. 6). Using a 60 $\times$ 60 grid with 3,600 force-estimation points, we analyzed the images with software capable of performing bead displacement measurements and force estimation (see supporting methods) and compared the characteristics of the three estimation algorithms.

For the neuronal growth cone, we quantified the bead distribution (Fig. 6 *A* top right) and applied a prior distribution of forces (Fig. 6 *A* bottom) to force estimation (Fig. 6 *B*, BTFE; Fig. 6 *C*, ridge; Fig. 6 *D*, lasso). The BTFE estimated large forces only in the cellular region, similar to the results with the synthetic data (Fig. 4), whereas the ridge regression estimated smaller forces overall. By comparison, the lasso regression showed sparse estimates of larger forces. The BTFE estimated large forces at growth-cone protrusions (Fig. 6 *B* bottom), which were not observed with the other algorithms (Fig. 6 *C* and *D* bottom). This result is consistent with the growth cone's ability to produce large traction forces to strain and elongate the neurite, which were correspondingly estimated as large forces by the BTFE algorithm.

We measured bead displacement from the bead images (Fig. 6 *E* top right) and used prior distributions (Fig. 6 *E* bottom) to estimate forces for the *Dictyostelium* cell. We found large differences in the force distribution, with the BTFE estimating large forces in the rear part of the cell for the migration direction (Fig. 6 *F*). The ridge and lasso estimations also estimated relatively large forces in the rear portion, which were comparable to those in the front portion, and large forces outside the cell in the rear (Fig. 6 *G* and *H*). Unlike growth cones, the *Dictyostelium* cell migrates by detaching the posterior adhesions from the substrate; hence, large forces were estimated in the rear.

For the fish epidermal keratocyte, using its bead distribution (Fig. 6 *I* top right) and prior distribution (Fig. 6 *I* bottom), the BTFE yielded prominent force estimations at both wing tips and the center-front part of lamellipodia boundaries in the direction of fish epidermal keratocyte movement (Fig. 6 *J*). The ridge estimation estimated weak forces over a wide area on both wings, extending to the cell's center (Fig. 6 *K*); however, unlike the BTFE, it did not estimate the center-front forces. The lasso estimation estimated large and uneven forces similar to those of the *Dictyostelium* cell (Fig. 6 *L*) and failed to estimate a small force in the center-front part of the cell.

DISCUSSION

In the present study, we introduced a Bayesian framework that uses cell boundary as a constraint to estimate cell traction force from small numbers of bead displacements. Because forces influence cell boundary, using cell boundary as a constraint is reasonable. However, cell boundary is too complex to formulate; previous force estimations have therefore not used it as a constraint. The ease of obtaining cell boundaries experimentally makes it an economical source of information to improve force-estimation accuracy. The present study showed that using LSM for boundary representation and MCF for cell transformation is suitable for formulating a cell boundary prior and reasonable force estimation with cell boundary constraints. The BTFE algorithm has a high degree of freedom in constraining forces. If the force prior in BTFE is a Gaussian or Laplace distribution unrelated to cell boundary, it corresponds to ridge or lasso regression, respectively. BTFE can introduce complex boundaries prior because of the high expression ability of the constraints. Simultaneously measuring actin filaments in the cell would enable us to extend the prior by introducing actin filament concentration, which contributes to force-estimation accuracy because the movement of actin filaments generates traction force. MCF can also compute a prior for 3D force estimation. In addition, a prior that better represents the cell force can be designed with other mathematical algorithms besides MCF.

Introducing BTFE required numerous hyperparameters, particularly for designing force priors. However, the large number of hyperparameters is not a significant problem because we can optimize most of them during force estimation. Rather, they are necessary to represent complex cell boundaries. The MCF simulation, which defines the direction prior, had hyperparameters for the diffusion coefficient of curvature and the elapsed time for cells to retract. These hyperparameters cannot be optimized by any algorithm. Nonetheless, the MCF is used to describe the definition of rough intracellular directions mathematically, which enables comprehensive calculation of the force directions at each cell boundary point. We used the EM algorithm to modify the magnitude and direction of the prior force by the MCF when estimating the force, followed by the final optimization of hyperparameters. Thus, hyperparameters associated with the MCF would not strongly affect the estimation results but might require some cell-dependent tuning. The accuracy in quantifying cell boundary and bead location substantially influences the estimation results more than the hyperparameters. In particular, accurate bead location is critical for evaluating substrate deformation. Therefore, we improved the accuracy of bead locations by developing software with automated processing and manual correction functions to detect bead locations from TFM images (supporting methods).

The ridge and lasso regressions estimate substrate stresses by fitting the model equation (Eq. 3) and assuming that forces occur where beads are present. This assumption is independent of bead density and is used even with high bead densities, making the force-estimation results for low bead densities unstable because of a greater variation in bead location (Figs. S3 and S4). However, BTFE can suppress stochasticity due to bead location because the cell boundary prior strongly constrains forces. Force estimation from TFM images also suggests the superiority of BTFE. A fish epidermal keratocyte has a horizontal boundary relative to the direction of movement and generates strong integrin-induced forces at both the left and right ends (52,53). All three methods produced similar force estimates (Fig. 6 *I–L*). Mechanics principles state that the combined force produced by the cell determines the direction of cell movement; thus, the wide lamellipodia at the front of the fish epidermal keratocyte must have small but broad spreading forces. However, the ridge and lasso regressions did not estimate such forces in the front lamellipodia, indicating that the accuracy of force estimation by these regressions was not reasonable. By contrast, BTFE provided force estimation at the lamellipodia site similar to that at other sites, demonstrating the significance of BTFE.

Traction force estimation using cell boundary can advance significantly when combined with other methods (54). Mean deformation metrics (MDM) estimate substrate deformation from changes in cell boundary, and accurate cell boundary extraction is critical for MDM and the Bayesian approach used in the present study, BTFE. Recent advances in deep learning have enabled accurate segmentation of cells in microscopic images (55,56). Combining the substrate deformation estimated by MDM with the cell-boundary-based force priors from the present study might enhance the

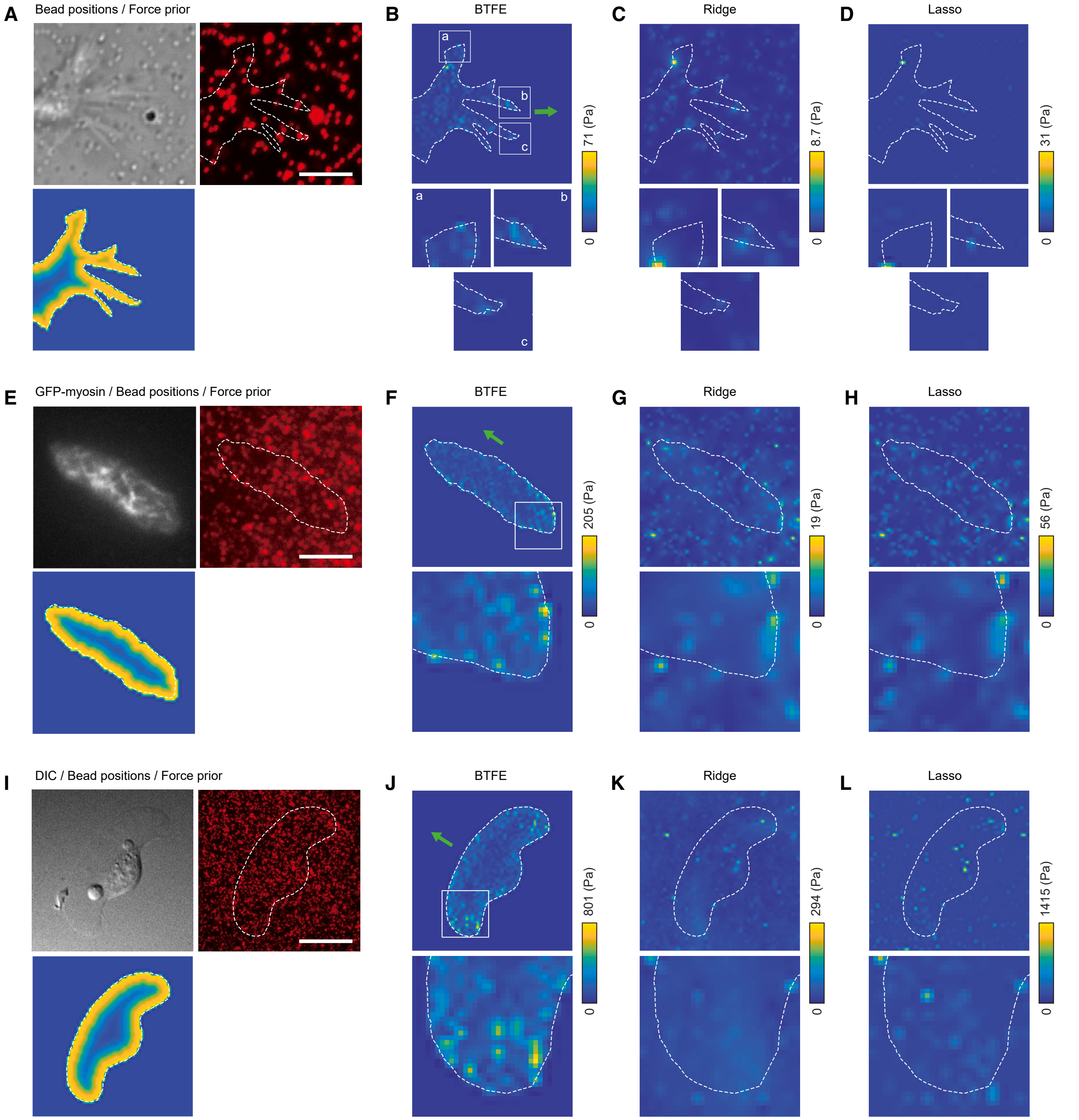

FIGURE 6 Comparison of force estimations using TFM images. (*A*) Images of neuronal growth-cone boundary and beads (*top*) and the corresponding Bayesian prior for force estimation (*bottom*). Cell boundary is represented by the white dashed line. (*B*) Bayesian force estimations using the beads in (*A*) (*top*) and focusing on pseudopods *a*, *b*, and *c* (*bottom*). The green arrow indicates cell migration direction. (*C* and *D*) Force estimation by ridge regression (*C*) and lasso regression (*D*) (*top*) and enlargements of the corresponding square areas to *a*, *b*, and *c* in (*B*) (*bottom*). Force estimation using ridge regression (*C*) and lasso regression (*D*) (*top*) with enlargements of corresponding areas to *a*, *b*, and *c* in (*B*) (*bottom*). (*E–H*) Force estimations for *Dictyostelium* cell. The lower panels show magnified views of the square regions, with all estimation results displayed on the same scale. (*I–L*) Force estimations for fish epidermal keratocyte. See supporting material for image preprocessing details. Scale bar, (*A*) 12 μm, (*E*) 5.53 μm, (I) 11.5 μm. For all calculations, we placed estimated force points on a 60 $\times$ 60 grid.

accuracy of cell traction force estimation. The integration of these technologies is an open issue that will be resolved in the future and is expected to contribute substantially to the development of mechanobiology.

Limitations of the study

As shown in Figs. 5 and S7, even when the algorithm proposed in the present study is used, the accuracy of force estimation

for cells with boundaries close to a circle is not significantly improved. In addition, good accuracy in force estimation might not be achieved for cells with many filopodia that are too thin to be quantified from images. If the unobservable tips of the filopodia deform the substrate, the distribution of forces would likely be calculated on the basis of the incorrect perception that the cell body caused the deformation. In addition, the bead position measurement is prone to noise, and suppressing noise as much as possible is effective in improving accuracy. Therefore, quantifying the cell boundary and bead position requires careful attention.

SUPPORTING MATERIAL

Supporting material can be found online at https://doi.org/10.1016/j.bpj.2023.10.032.

AUTHOR CONTRIBUTIONS

Y.S. designed the project. R.F., S K., K.I., and Y.S. developed the computational model and data analyses. N.I., C.O., and Y.I. performed experiment and data preprocessing. R.F., Y.I., K.I., N.I., and Y.S. prepared the manuscript.

ACKNOWLEDGMENTS

We thank T. Yamada, K. Kunida, T. Sakurai, and T. J. Kobayashi for helpful discussion and technical comments. S.K. was affiliated with Nara Institute of Science and Technology at the time of the work and is currently affiliated with Advanced Telecommunications Research Institute International, Japan.This work was supported by the Japan Agency for Medical Research and Development (AMED) (grant number JP22gm0810011 for N.I. and Y.S.), JSPS KAKENHI, Japan (grant number 23H04707 for Y.S.), and NAIST Next Generation Interdisciplinary Research Project (Y.S.).

DECLARATION OF INTERESTS

The authors declare no competing interests.

REFERENCES

1. Calandra, T., and R. Bucala. 1997. Macrophage migration inhibitory factor (MIF): a glucocorticoid counter-regulator within the immune system. *Crit. Rev. Immunol.* 17:77–88.
2. Luster, A. D., R. Alon, and U. H. von Andrian. 2005. Immune cell migration in inflammation: present and future therapeutic targets. *Nat. Immunol.* 6:1182–1190.
3. Tessier-Lavigne, M., and C. S. Goodman. 1996. The molecular biology of axon guidance. *Science.* 274:1123–1133.
4. Ma, Q., D. Jones, ..., T. A. Springer. 1998. Impaired B-lymphopoiesis, myelopoiesis, and derailed cerebellar neuron migration in CXCR4- and SDF-1-deficient mice. *Proc. Natl. Acad. Sci. USA.* 95:9448–9453.
5. Dickson, B. J. 2002. Molecular mechanisms of axon guidance. *Science.* 298:1959–1964.
6. Charron, F., and M. Tessier-Lavigne. 2005. Novel brain wiring functions for classical morphogens: a role as graded positional cues in axon guidance. *Development.* 132:2251–2262.
7. Kleeberger, W., G. S. Bova, ..., D. M. Berman. 2007. Roles for the stem cell–associated intermediate filament nestin in prostate cancer migration and metastasis. *Cancer Res.* 67:9199–9206.
8. O'Hayre, M., C. L. Salanga, ..., S. J. Allen. 2008. Chemokines and cancer: migration, intracellular signalling and intercellular communication in the microenvironment. *Biochem. J.* 409:635–649.
9. Tan, J. L., J. Tien, ..., C. S. Chen. 2003. Cells lying on a bed of microneedles: An approach to isolate mechanical force. *Proc. Natl. Acad. Sci. USA.* 100:1484–1489.
10. Dembo, M., T. Oliver, ..., K. Jacobson. 1996. Imaging the traction stresses exerted by locomoting cells with the elastic substratum method. *Biophys. J.* 70:2008–2022.
11. Chan, C. E., and D. J. Odde. 2008. Traction dynamics of filopodia on compliant substrates. *Science.* 322:1687–1691.
12. Toriyama, M., S. Kozawa, ..., N. Inagaki. 2013. Conversion of a signal into forces for axon outgrowth through Pak1-mediated Shootin1 phosphorylation. *Curr. Biol.* 23:529–534.
13. Stricker, J., B. Sabass, ..., M. L. Gardel. 2010. Optimization of traction force microscopy for micron-sized focal adhesions. *J. Phys. Condens. Matter.* 22, 194104.
14. Gardel, M. L., B. Sabass, ..., C. M. Waterman. 2008. Traction stress in focal adhesions correlates biphasically with actin retrograde flow speed. *J. Cell Biol.* 183:999–1005.
15. Lemmon, C. A., N. J. Sniadecki, ..., C. S. Chen. 2005. Shear force at the cell-matrix interface: enhanced analysis for microfabricated post array detectors. *Mech. Chem. Biosyst.* 2:1–16.
16. Landau, L. D., and E. M. Lifshitz. 1970. Theory of Elasticity. Second Revised and Enlarged. Pergamon Press Ltd.
17. Sabass, B., M. L. Gardel, ..., U. S. Schwarz. 2008. High resolution traction force microscopy based on experimental and computational advances. *Biophys. J.* 94:207–220.
18. Soiné, J. R. D., C. A. Brand, ..., U. S. Schwarz. 2015. Model-based traction force microscopy reveals differential tension in cellular actin bundles. *PLoS Comput. Biol.* 11, e1004076.
19. Bornschlögl, T., S. Romero, ..., P. Bassereau. 2013. Filopodial retraction force is generated by cortical actin dynamics and controlled by reversible tethering at the tip. *Proc. Natl. Acad. Sci. USA.* 110:18928–18933.
20. Stubb, A., R. F. Laine, ..., J. Ivaska. 2020. Fluctuation-based super-resolution traction force microscopy. *Nano Lett.* 20:2230–2245.
21. Kushch, V. I. 2019. Elastic ellipsoidal inhomogeneity with imperfect interface: Complete displacement solution in terms of ellipsoidal harmonics. *Int. J. Solid Struct.* 166:83–95.
22. Jasiuk, I., P. Y. Sheng, and E. Tsuchida. 1997. A spherical inclusion in an elastic half-space under shear. *J. Appl. Mech.* 64:471–479.
23. Delfani, M. R., and M. Latifi Shahandashti. 2017. Elastic field of a spherical inclusion with non-uniform eigenfields in second strain gradient elasticity. *Proc. R. Soc. A A.* 473, 20170254.
24. Dembo, M., and Y.-L. Wang. 1999. Stresses at the cell-to-substrate interface during locomotion of fibroblasts. *Biophys. J.* 76:2307–2316.
25. Butler, J. P., I. M. Tolić-Nørrelykke, ..., J. J. Fredberg. 2002. Traction fields, moments, and strain energy that cells exert on their surroundings. *Am. J. Physiol. Cell Physiol.* 282:595–605.
26. Li, D., H. Colin-York, ..., M. Fritzsche. 2021. Astigmatic traction force microscopy (aTFM). *Nat. Commun.* 12:2168.
27. Barbieri, L., H. Colin-York, ..., M. Fritzsche. 2021. Two-dimensional TIRF-SIM–traction force microscopy (2D TIRF-SIM-TFM). *Nat. Commun.* 12:2169.
28. Yang, Z., J.-S. Lin, ..., J. H.-C. Wang. 2006. Determining substrate displacement and cell traction fields—a new approach. *J. Theor. Biol.* 242:607–616.
29. Han, S. J., Y. Oak, ..., G. Danuser. 2015. Traction microscopy to identify force modulation in subresolution adhesions. *Nat. Methods.* 12:653–656.

30. Suñé-Auñón, A., A. Jorge-Peñas, …, A. Muñoz-Barrutia. 2016. L1-regularized reconstruction for traction force microscopy. *In* 2016 IEEE 13th Int Symposium Biomed Imaging, pp. 140–144.

31. Suñé-Auñón, A., A. Jorge-Peñas, …, A. Muñoz-Barrutia. 2017. Full L1-regularized traction force microscopy over whole cells. *BMC Bioinf.* 18:365.

32. Ponti, A., M. Machacek, …, G. Danuser. 2004. Two distinct actin networks drive the protrusion of migrating cells. *Science.* 305:1782–1786.

33. Pollard, T. D., and G. G. Borisy. 2003. Cellular motility driven by assembly and disassembly of actin filaments. *Cell.* 112:453–465.

34. Forscher, P., and S. J. Smith. 1988. Actions of cytochalasins on the organization of actin filaments and microtubules in a neuronal growth cone. *J. Cell Biol.* 107:1505–1516.

35. Lin, C. H., E. M. Espreafico, …, P. Forscher. 1996. Myosin drives retrograde F-actin flow in neuronal growth cones. *Neuron.* 16:769–782.

36. Pantaloni, D., C. Le Clainche, and M.-F. Carlier. 2001. Mechanism of actin-based motility. *Science.* 292:1502–1506.

37. Janetopoulos, C., L. Ma, …, P. A. Iglesias. 2004. Chemoattractant-induced phosphatidylinositol 3,4,5-trisphosphate accumulation is spatially amplified and adapts, independent of the actin cytoskeleton. *Proc. Natl. Acad. Sci. USA.* 101:8951–8956.

38. Li, J., D. Han, and Y.-P. Zhao. 2014. Kinetic behaviour of the cells touching substrate: the interfacial stiffness guides cell spreading. *Sci. Rep.* 4:3910.

39. Lemmon, C. A., and L. H. Romer. 2010. A predictive model of cell traction forces based on cell geometry. *Biophys. J.* 99:78–80.

40. Atia, L., D. Bi, …, J. J. Fredberg. 2018. Geometric constraints during epithelial jamming. *Nat. Phys.* 14:613–620.

41. Helfrich, W. 1973. Elastic properties of lipid bilayers: theory and possible experiments. *Z. Naturforsch. C Biosci.* 28:693–703.

42. Helfrich, W. 1974. The size of bilayer vesicles generated by sonication. *Phys. Lett.* 50:115–116.

43. Helfrich, W. 1974. Blocked lipid exchange in bilayers and its possible influence on the shape of vesicles. *Z. Naturforsch. C Biosci.* 29C:510–515.

44. Osher, S., and J. A. Sethian. 1988. Fronts propagating with curvature-dependent speed: Algorithms based on Hamilton-Jacobi formulations. *J. Comput. Phys.* 79:12–49.

45. Machacek, M., and G. Danuser. 2006. Morphodynamic profiling of protrusion phenotypes. *Biophys. J.* 90:1439–1452.

46. Huang, Y., C. Schell, …, B. Sabass. 2019. Traction force microscopy with optimized regularization and automated Bayesian parameter selection for comparing cells. *Sci. Rep.* 9:539.

47. Hore, A., and D. Ziou. 2010. Image quality metrics: PSNR vs. SSIM. *2010 20th Int Conf Pattern Recognit* 2366–2369.

48. Wang, Y.-L., and R. J. Pelham. 1998. Preparation of a flexible, porous polyacrylamide substrate for mechanical studies of cultured cells. *Methods Enzymol.* 298:489–496.

49. Bridgman, P. C., S. Dave, …, R. S. Adelstein. 2001. Myosin IIB is required for growth cone motility. *J. Neurosci.* 21:6159–6169.

50. Okimura, C., Y. Sakumura, …, Y. Iwadate. 2018. Sensing of substratum rigidity and directional migration by fast-crawling cells. *Phys. Rev. E.* 97, 052401.

51. Sonoda, A., C. Okimura, and Y. Iwadate. 2016. Shape and area of keratocytes are related to the distribution and magnitude of their traction forces. *Cell Struct. Funct.* 41:33–43.

52. Lee, J., M. Leonard, …, K. Jacobson. 1994. Traction forces generated by locomoting keratocytes. *J. Cell Biol.* 127:1957–1964, –1964.

53. Doyle, A., W. Marganski, and J. Lee. 2004. Calcium transients induce spatially coordinated increases in traction force during the movement of fish keratocytes. *J. Cell Sci.* 117:2203–2214.

54. Stout, D. A., E. Bar-Kochba, …, C. Franck. 2016. Mean deformation metrics for quantifying 3D cell–matrix interactions without requiring information about matrix material properties. *Proc. Natl. Acad. Sci. USA.* 113:2898–2903.

55. Al-Kofahi, Y., A. Zaltsman, …, M. Rusu. 2018. A deep learning-based algorithm for 2-D cell segmentation in microscopy images. *BMC Bioinf.* 19:365.

56. Majurski, M., P. Manescu, …, P. Bajcsy. 2019. Cell image segmentation using generative adversarial networks, transfer learning, and augmentations. *In* 2019 IEEE/CVF Conference on Computer Vision and Pattern Recognition Workshops, pp. 1114–1122.

Biophysical Journal, Volume *122*

Supplemental information

Bayesian traction force estimation using cell boundary-dependent force priors

Ryosuke Fujikawa, Chika Okimura, Satoshi Kozawa, Kazushi Ikeda, Naoyuki Inagaki, Yoshiaki Iwadate, and Yuichi Sakumura

Supplemental Methods

1. Virtual cell deformation by mean curvature flow

The changes in cell shape using LSF can be described by the Hamilton–Jacobi equation:

$$\frac{\partial \phi(\mathbf{x},t)}{\partial t} = F(\mathbf{x},t;\phi)|\nabla\phi(\mathbf{x},t)|\,, \tag{S1}$$

where $F(\mathbf{x},t;\phi)$ and $|\nabla\phi(\mathbf{x},t)|$ represent the velocity of edge movement and the length of the normal vector at each edge point, respectively. Because edge points are sets that satisfy $\phi(\mathbf{x},t) = 0$, increasing $\phi(\mathbf{x},t)$ to $\phi(\mathbf{x},t) > 0$ implies cell retraction. Thus, the cell retracts when the velocity $F(\mathbf{x},t;\phi)$ is positive. We defined the velocity $F(\mathbf{x},t;\phi)$ using the mean curvature flow (MCF) algorithm (1). The MCF algorithm diffuses and homogenizes the curvature of a curve or surface. The change of the average curvature at the edge point $\mathbf{x}$ at time t, $\kappa(\mathbf{x},t;\ \phi)$, is defined as

$$\kappa(\mathbf{x},t;\ \phi) = -\frac{1}{2}\nabla\cdot\left(\frac{\nabla\phi(\mathbf{x},t)}{|\nabla\phi(\mathbf{x},t)|}\right), \tag{S2}$$

which is negative at protruding edges and reduces the curvature. We then defined the edge movement velocity as

$$F(\mathbf{x},t;\phi) = -D\kappa(\mathbf{x},t;\ \phi)\,, \tag{S3}$$

where D is the location-independent diffusion coefficient. We substituted Eq. (S3) into Eq. (S1) and moved the edges until time $t = \tau$, when the curvature was sufficiently diffused. In this study, τ was set such that $\tau > 0.1[Cell\ area]\ /\ D$ on the basis of 10% of the cell area. This condition increases τ to diffuse curvature for cells with large areas. We used MATLAB (Mathworks) to simulate cell deformation and Mitchell's program package (2) to calculate the MCF.

2. EM algorithm for estimating forces and hyperparameters

We used the EM algorithm to maximize the log posterior probability distribution $\log P(\{\theta_n,s_n\}_{n=1}^N|\mathbf{u}_{obs})$ of the angle and magnitude $\{\theta_n,s_n\}$ $(n = 1,\cdots,N)$ of each force for the bead displacement observation $\mathbf{u}_{obs}$, and simultaneously approximate the posterior probability distribution of traction force $\mathbf{f}$, $P(\mathbf{f}|\mathbf{u}_{obs},\{\theta_n,s_n\}_{n=1}^N)$. Using the likelihood of bead displacement,

$$P(\mathbf{u}_{obs}|\{\theta_n,s_n\}_{n=1}^N) = \frac{P(\mathbf{u}_{obs}|\boldsymbol{f})\times P(\boldsymbol{f}|\{\theta_n,s_n\}_{n=1}^N)}{P(\boldsymbol{f}|\mathbf{u}_{obs},\{\theta_n,s_n\}_{n=1}^N)}\,,$$

where $P(\mathbf{f}|\mathbf{u}_{obs},\{\theta_n,s_n\}_{n=1}^N)$ is $\mathbf{f}$ posterior, we obtained posterior probability distributions for the angle and magnitude of the forces:

$$P(\{\theta_n,s_n\}_{n=1}^N|\mathbf{u}_{obs}) \propto P(\mathbf{u}_{obs}|\{\theta_n,s_n\}_{n=1}^N)\times P(\{\theta_n,s_n\}_{n=1}^N)\,. \tag{S4}$$

Therefore, the log posterior probability distribution of $\{\theta_n,s_n\}$ became

$$\log P(\{\theta_n,s_n\}_{n=1}^N|\mathbf{u}_{obs}) = \log P(\mathbf{u}_{obs}|\{\theta_n,s_n\}_{n=1}^N) + \log P(\{s_n\}_{n=1}^N) + const., \tag{S5}$$

where we included the uninformative direction prior $P(\{\theta_n\})$ in the third term. To estimate the hyperparameters $\{\theta_n,s_n\}$ from Eq. (S5) for the bead displacement observation $\mathbf{u}_{obs}$, we introduced the EM algorithm while obtaining an approximate function to estimate $\mathbf{f}$.

The sum of the first and second terms in Eq. (S5) determines the maximum log-posterior. For the first term, we used a probability density function $q(\mathbf{f})$ that approximates $\mathbf{f}$ posterior $P(\mathbf{f}|\mathbf{u}_{obs},\{\theta_n,s_n\}_{n=1}^N)$ and transformed the expression as follows:

$$\log P(\mathbf{u}_{obs}|\{\theta_n, s_n\}_{n=1}^N) = \int_{\mathbb{R}^{2N}} q(\mathbf{f}) \log \frac{P(\mathbf{u}_{obs}|\mathbf{f}) \times P(\mathbf{f}|\{\theta_n, s_n\}_{n=1}^N)}{P(\mathbf{f}|\mathbf{u}_{obs}, \{\theta_n, s_n\}_{n=1}^N)} d\mathbf{f}$$
$$= -\int_{\mathbb{R}^{2N}} q(\mathbf{f}) \log \frac{q(\mathbf{f})}{P(\mathbf{u}_{obs}|\mathbf{f}) \times P(\mathbf{f}|\{\theta_n, s_n\}_{n=1}^N)} d\mathbf{f}$$
$$+ \int_{\mathbb{R}^{2N}} q(\mathbf{f}) \log q(\mathbf{f})\, d\mathbf{f}$$
$$- \int_{\mathbb{R}^{2N}} q(\mathbf{f}) \log P(\mathbf{f}|\mathbf{u}_{obs}, \{\theta_n, s_n\}_{n=1}^N)\, d\mathbf{f} \ . \quad \text{(S6)}$$

The integral in the first term of Eq. (S6) is non-negative and is known as the Kullback–Leibler divergence; it is zero when $q(\mathbf{f}) = P(\mathbf{u}_{obs}|\mathbf{f}) \times P(\mathbf{f}|\{\theta_n, s_n\}_{n=1}^N)$. On the basis of our EM algorithm, we alternately update the **f** posterior approximation $q(\mathbf{f})$ and $\{\theta_n, s_n\}_{n=1}^N$ to maximize Eq. (S5) while satisfying this condition. Let the value of $\{\theta_n, s_n\}$ at the k-th update be written as $\{\theta_n^k, s_n^k\}$. In the expectation step (E-step), $\{\theta_n^k, s_n^k\}$ is fixed and $q(\mathbf{f})$ is approximated by a Gaussian distribution:

$$q(\mathbf{f}|\boldsymbol{\mu}_f^k, \Sigma_f) \propto \exp\left\{-\frac{1}{2}(\mathbf{f} - \boldsymbol{\mu}_f^k)^T \Sigma_f^{-1} (\mathbf{f} - \boldsymbol{\mu}_f^k)\right\} . \quad \text{(S7)}$$

The mean $\boldsymbol{\mu}_f$ and covariance Σ_f of the parameters are the values for which the first term in Eq. (S6) is zero; we therefore obtained

$$\boldsymbol{\mu}_f^k = \Sigma_f(\alpha G^T \mathbf{u}_{obs} + \beta \mathbf{f}_{\mu}\{\theta_n^k, s_n^k\})$$
$$\Sigma_f = (\alpha G^T G + \beta I_{2N})^{-1} ,$$

where the parameters and the variables are explained in the text. In the maximization step (M-step), when the approximated distribution $q(\mathbf{f}|\boldsymbol{\mu}_f^k, \Sigma_f)$ is substituted into the log-likelihood (Eq. (S6)), the k-th log-posterior distribution (Eq. (S5)) becomes

$$Q^k(\{\theta_n, s_n\}_{n=1}^N) = -\int_{\mathbb{R}^{2N}} q(\mathbf{f}|\boldsymbol{\mu}_f^k, \Sigma_f) \log P(\mathbf{f}|\mathbf{u}_{obs}, \{\theta_n, s_n\}_{n=1}^N)\, d\mathbf{f} - \gamma \sum_{n=1}^{N} |s_n| \ . \quad \text{(S8)}$$

We found $\{\theta_n^k, s_n^k\}$ that maximizes this distribution. By repeating the E- and M-steps, we incrementally computed

$$\mathbf{f}_{est} = \arg\max_{\mathbf{f}} q(\mathbf{f}|\boldsymbol{\mu}_f^k, \Sigma_f)$$
$$\{\theta_n^{k+1}, s_n^{k+1}\} = \arg\max_{\{\theta_n, s_n\}} Q^k(\{\theta_n, s_n\}_{n=1}^N) \ . \quad \text{(S9)}$$

We then adopted $\{\theta_n, s_n\}_{n=1}^N$ when $\mathbf{f}_{est}$ was sufficiently convergent. The initial conditions were $\theta_n^0 = 0$, $s_n^0 = 1$, $(n = 1, \dots, N)$. A conceptual diagram illustrating the prior design characterized by hyperparameters and the EM algorithm is provided in **Fig. S1***B*.

3. Software for quantifying cells and estimating forces

We developed MATLAB software for force estimation using Bayesian traction force estimation (BTFE), ridge, and lasso regressions. The software, available at https://github.com/sakulab-software/Bayesian_Force_Estimation, provides a graphical interface for viewing and manipulating microscopic images. Users can easily perform image preprocessing and extract cellular regions using intuitive tools. The software requires the MATLAB main unit and Image Processing Toolbox (version R2021a or later) and supports computational efficiency through the Parallel Computing Toolbox.

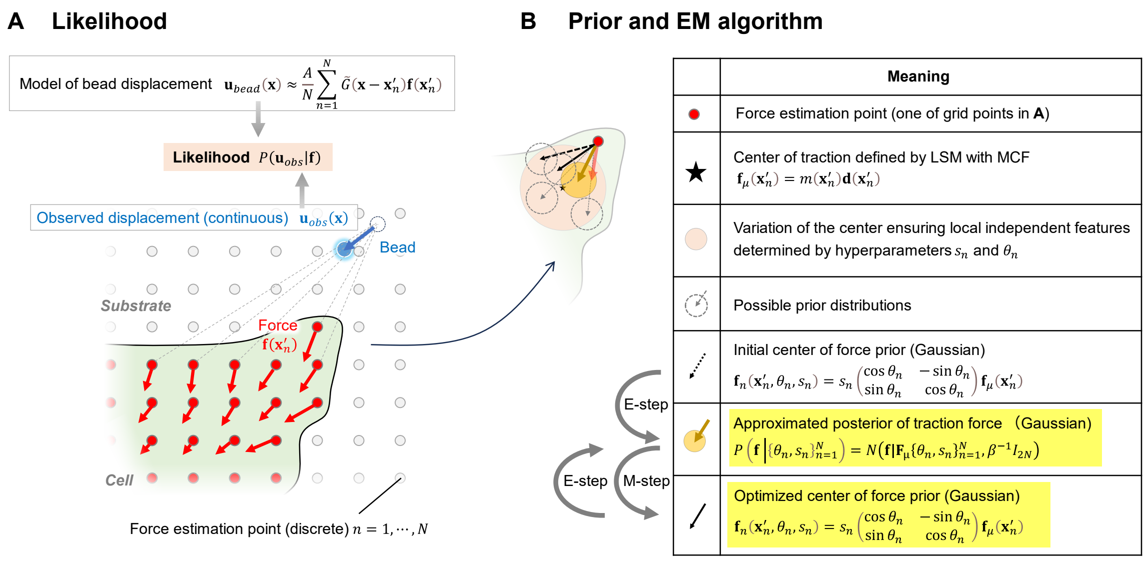

Figure S1. Conceptual diagram of likelihood, prior, and approximation algorithm
(A) The likelihood is calculated on the basis of the difference between the observed continuous bead displacement $\mathbf{u}_{obs}$ (blue vector) and the model displacement $\mathbf{u}_{bead}$ resulting from the force $\mathbf{f}$ arranged on a grid. The force $\mathbf{f}$ at each grid point (red arrow) is the estimation target.
(B) The relationship between the design of the prior and the EM algorithm used for approximating the force $\mathbf{f}$. Initially, the center of the force prior ($\mathbf{f}_\mu$) at each grid point (red point) is roughly determined from the cell boundary in terms of magnitude (m) and direction ($\mathbf{d}$) (black star; refer to the Methods section in the main text); $\mathbf{f}_\mu$ is locally scaled (s) and rotated (θ) at each grid point to create the actual center of the prior ($\mathbf{f}_n$). Candidates for $\mathbf{f}_n$ exist within the pink filled circle, and their surrounding area (gray dashed edge circle) represents the distribution of the force prior. Parameters s and θ are iteratively adjusted to maximize the peak of the posterior distribution of $\mathbf{f}$ (maximum a posteriori; MAP, gold arrow). Initially, an initial value (dotted arrow) is set, and the peak $\mathbf{f}$ of $\mathbf{f}$'s posterior distribution is calculated (E-step, Eq. (S7)). With the force fixed at that peak, s and θ are then optimized using MAP estimation (solid black arrow; M-step, Eq. (S8)). This process is repeated for each grid point until convergence.

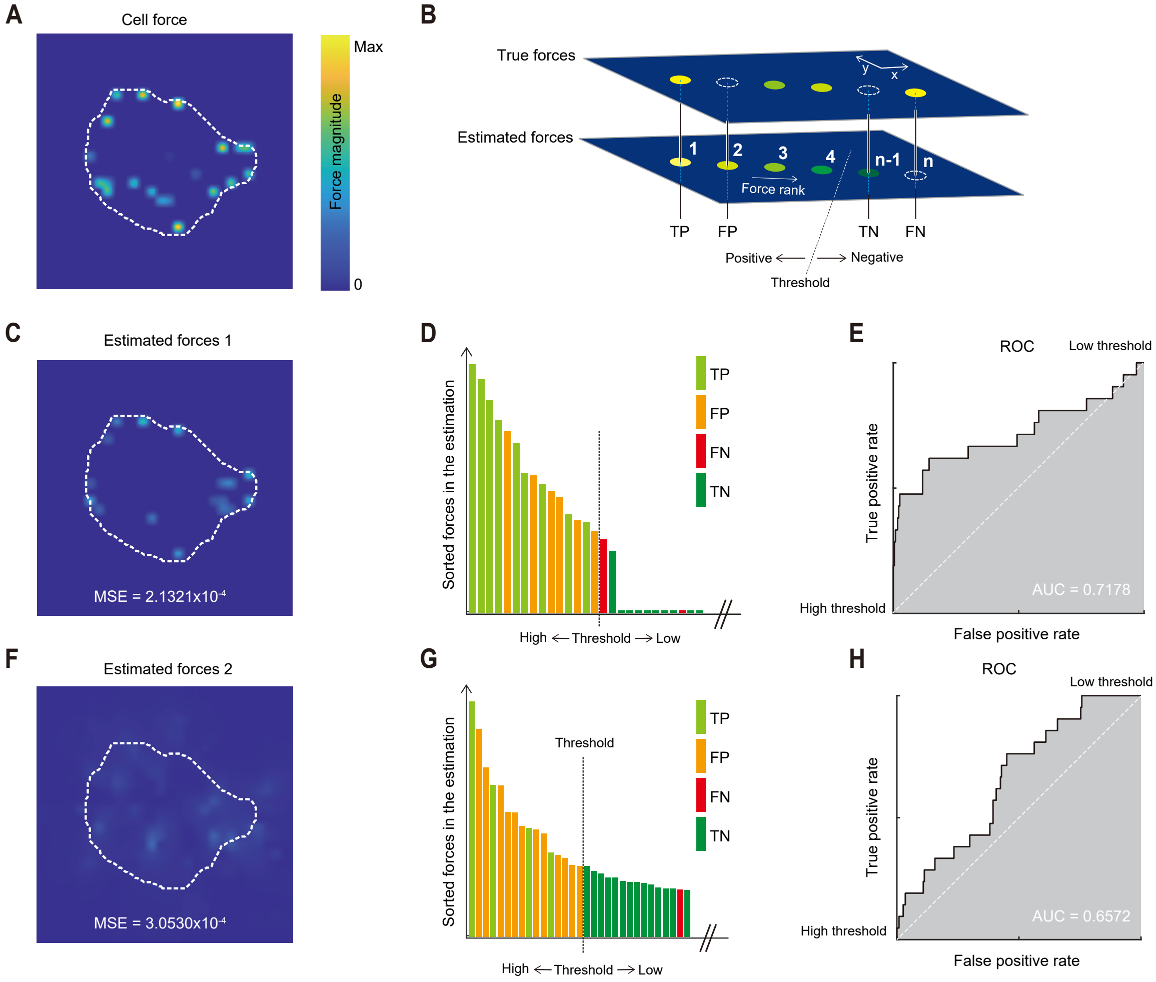

Figure S2. Derivation of ROC curves for evaluating force estimation accuracy.
(A) Force distribution generated by the model cell, with the force magnitude represented by a color bar.
(B) Threshold value used to determine positive and negative force detection. A force greater than the threshold was considered positive, whereas one less than the threshold was deemed negative. Determination of true or false was based on the presence or absence, respectively, of model cell forces. We calculated the rates of true positives and false positives for each threshold and drew ROC curves.
(C) Sample image of the forces estimated by the Bayes approach.
(D) Bar graph of the forces in (C), sorted by magnitude, with the four labels displayed in different colors.
(E) ROC curve derived from (D). The true positive rate is calculated as TP/(TP + FN), and the false positive rate is determined as FP/(FP + TN). Both the true positive rate and the false positive rate are low when the thresholds are large because only large estimated forces are positive. Decreasing the threshold raises the false positive force due to noise while capturing more true positive force.
(F–H) Same as (C–E) but with the forces estimated by ridge regression.

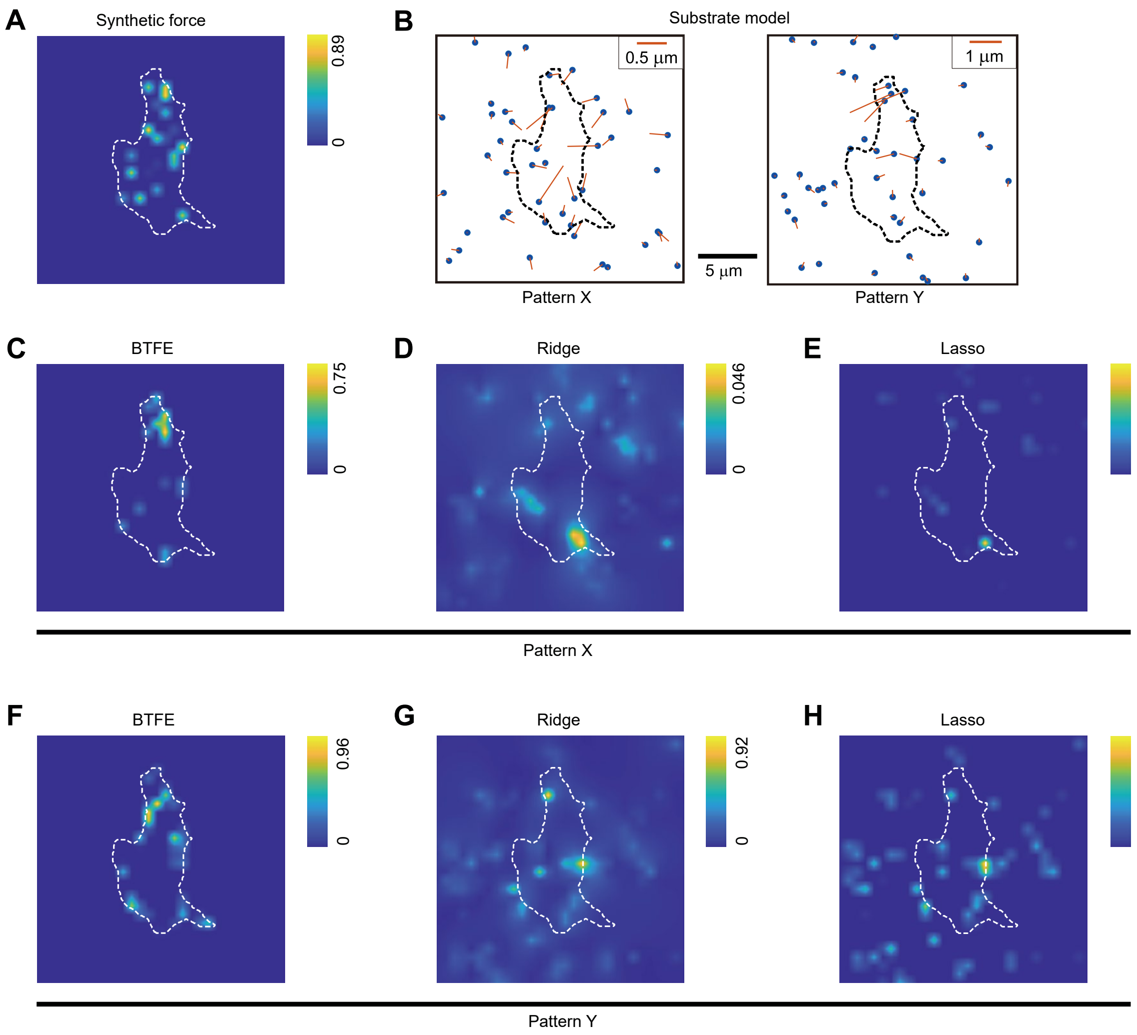

Figure S3. Dependence of force estimation on bead location for bead density of 0.4 beads/µm^2.

(A) The synthetic force in Fig. 4*A*, redisplayed for visual comparison with estimated forces.

(B) Two patterns of bead locations randomly spread at 0.4 bead/µm^2 on the substrate, different from Fig. 4*B*.

(C–E) Comparison of estimation results from the three different algorithms (C, Bayes; D, ridge; E, lasso) for the case of pattern X bead allocation.

(F–H) Estimation results for the case of pattern Y bead allocation.

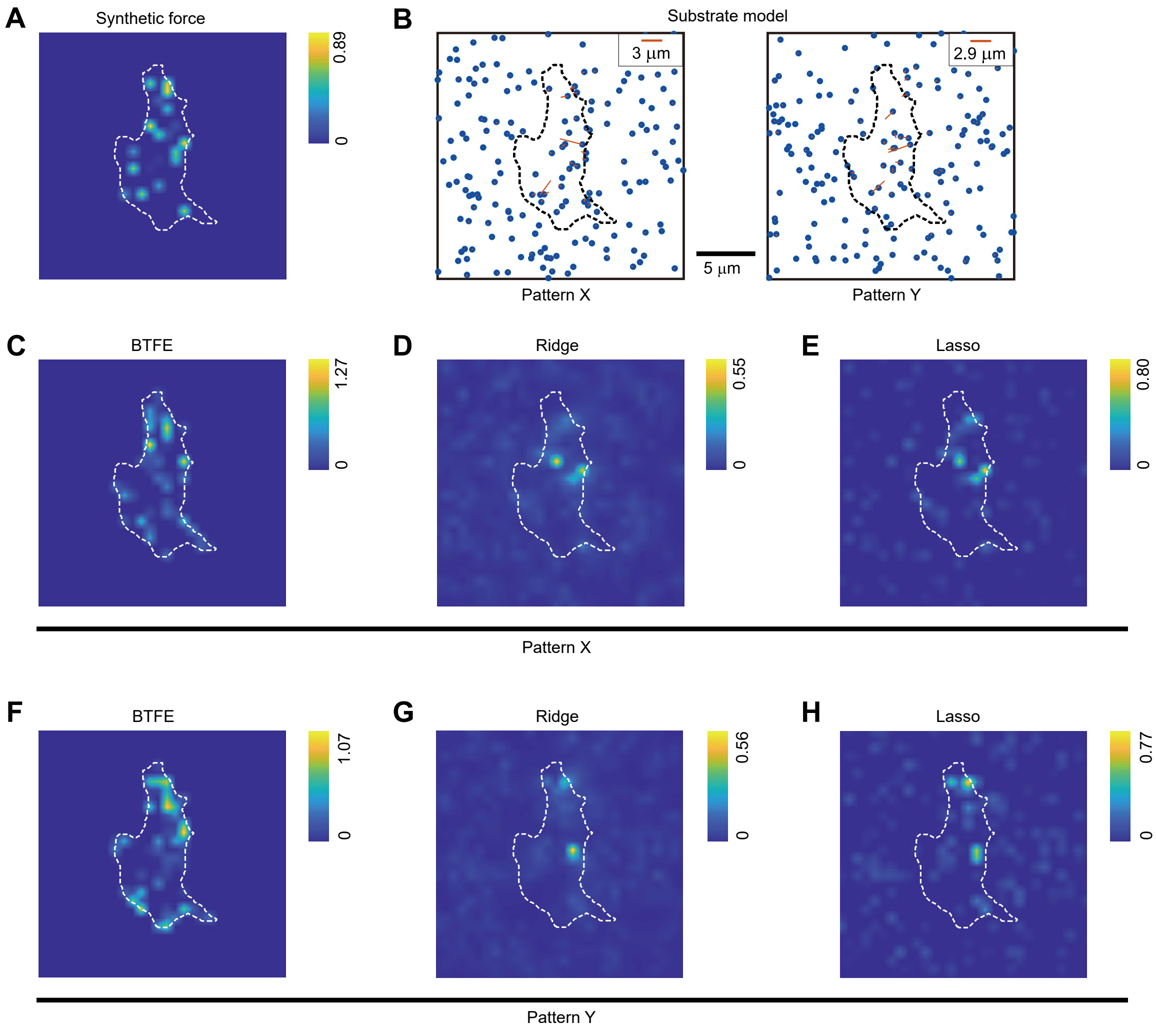

Fig. S4. Dependence of force estimation on bead location for bead density of 1.6 beads/μm^2.

(A) The synthetic force in Fig. 4*A*, redisplayed for visual comparison with estimated forces. (B) Two patterns of bead positions randomly spread at 1.6 bead/μm^2 on the substrate, different from Fig. 4*B*. (C–E) Comparison of estimation results from the three different algorithms (C, Bayes; D, ridge; E, lasso) for the case of pattern X bead allocation.

(F–H) Estimation results for the case of pattern Y bead allocation.

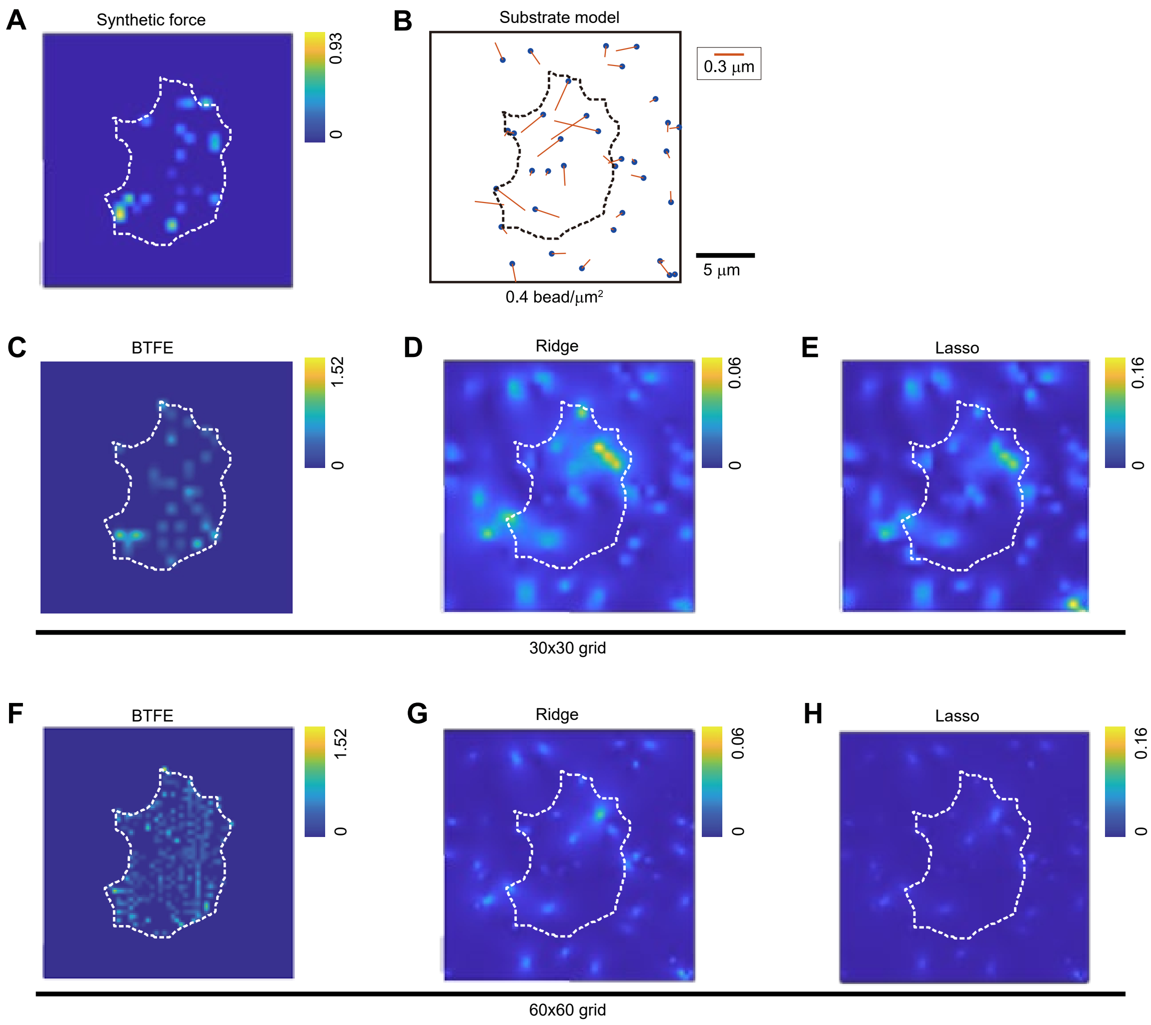

Figure S5. Dependency of force estimation on grid density.
(A) The synthetic force.
(B) Bead positions randomly distributed at 0.4 bead/μm^2 on the substrate.
(C)–(E) Estimation results calculated by BTFE, ridge, and lasso, respectively, using the default grid density (30×30).
(F)–(H) Corresponding results using a high grid density (60×60).

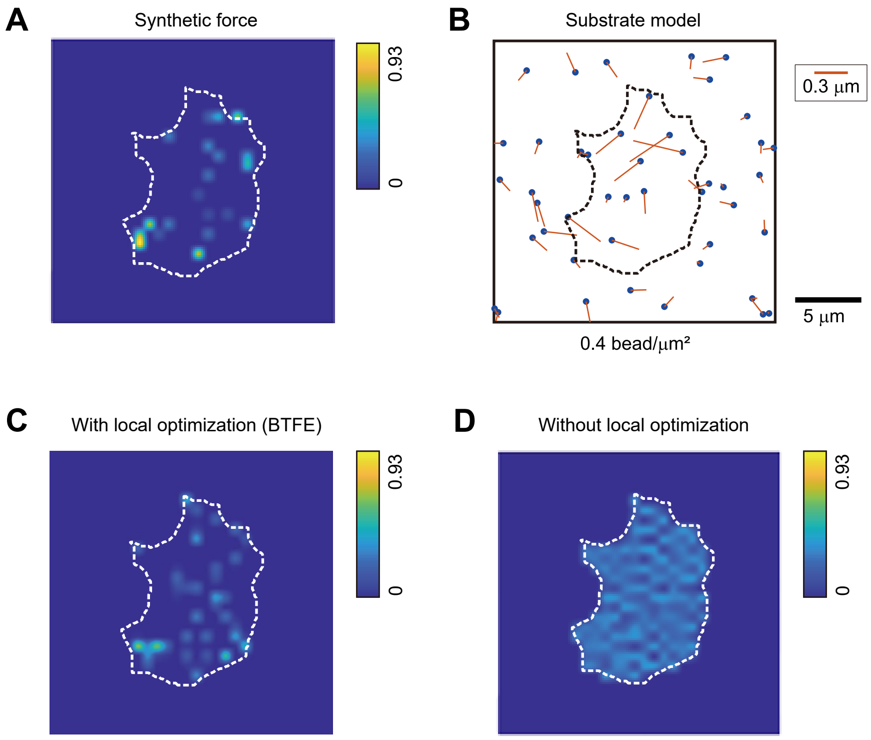

Figure S6. Effect of local optimizations to force prior on force estimation accuracy.
(A) Assumed artificial force generation based on the boundary of the HT1080 cell.
(B) Bead displacement resulting from the force in (A).
(C) Estimation results using locally optimized force priors in BTFE.
(D) Estimation results using force priors without local optimization.

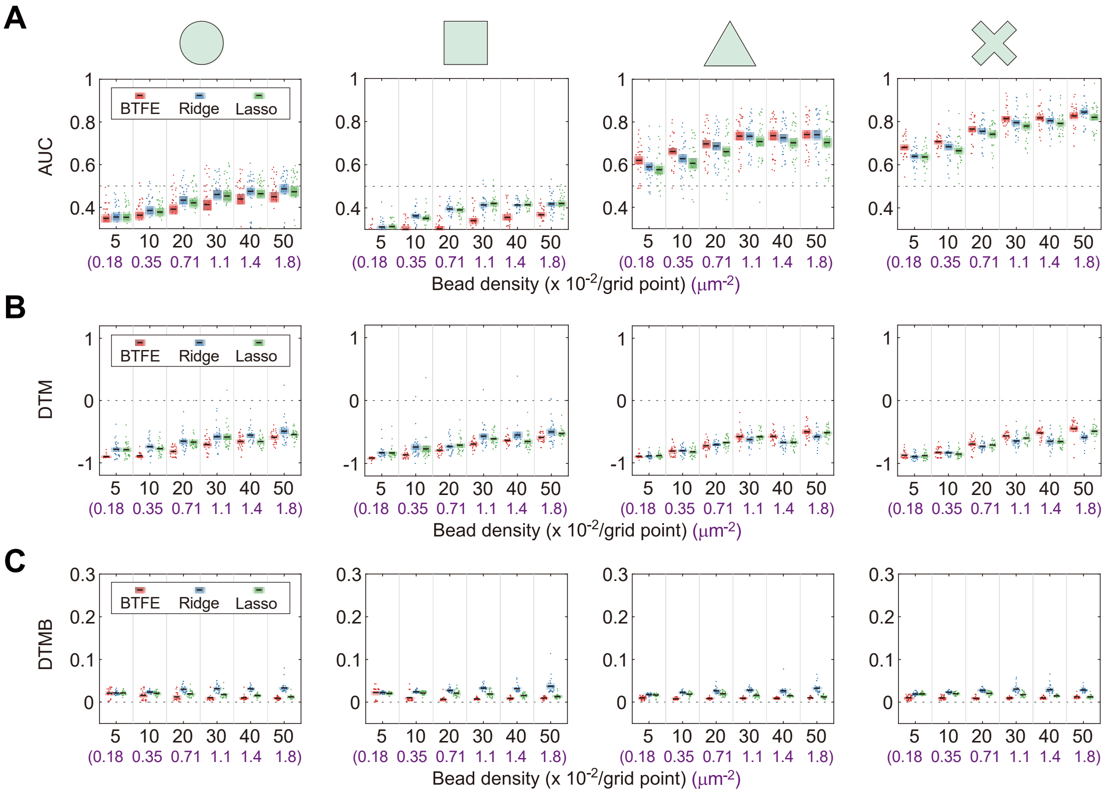

Figure S7. Relationship between geometric boundaries and force estimation accuracy. From left to right, we evaluated force estimation for cells assumed to have the boundaries of a perfect circle, square, equilateral triangle, and X-shape. Metrics used for evaluation include AUC (A), DTM (B), and DTMB (C), following the format outlined in **Figs. 5***D–F*.

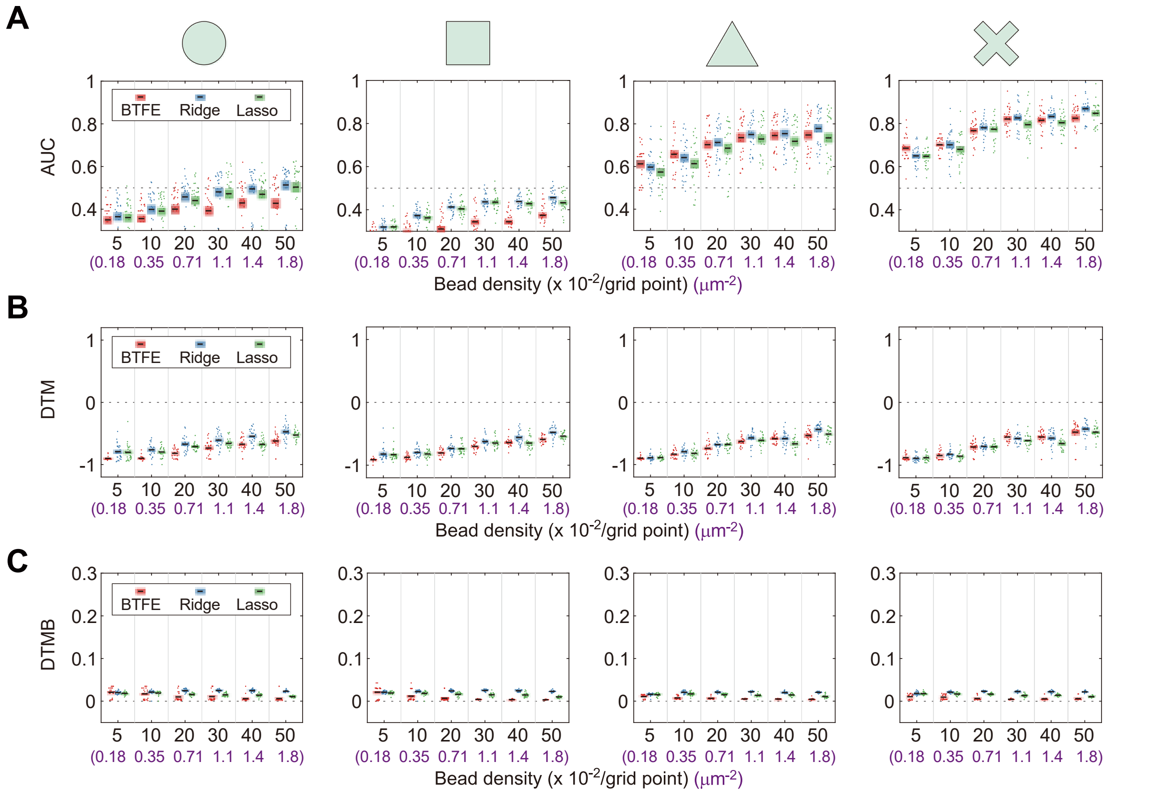

Figure S8. Relationship between geometric boundaries and force estimation accuracy in the absence of bead displacement noise.

From left to right, the force estimation accuracy is presented for cells with boundaries of a perfect circle, square, equilateral triangle, and X-shape, using noise-free bead displacement data. Following the format of **Figs. 5***D–F*, the force estimation is displayed in terms of (A) AUC, (B) DTM, and (C) DTMB.

Supplementary references

1. Colding, T.H., W.P. Minicozzi, and E.K. Pedersen. 2015. Mean curvature flow. *B Am Math Soc*. 52:297–333.

2. Mitchell, I.M. 2008. The flexible, extensible and efficient toolbox of level set methods. *J Sci Comput*. 35:300–329.
